# Wild-type and resistance-breaking strains of tomato spotted wilt virus differentially upregulate the immunosuppressive epoxyoctadecamonoenoic acid biosynthesis of its insect vector, Frankliniella occidentalis

**DOI:** 10.1099/jgv.0.002175

**Published:** 2025-11-07

**Authors:** Niayesh Shahmohammadi, Falguni Khan, Donghee Lee, Daehong Lee, Yonggyun Kim

**Affiliations:** 1 School of Life Sciences, College of Life Sciences and Engineering, Gyeongkuk National University, Andong 36729, South Korea; 2 Industry Academy Cooperation Foundation, Gyeongkuk National University, Andong 36729, South Korea; 3 Yeongyang Pepper Research Institute, Gyeongsangbukdo Agricultural Research and Extension Services, Yeongyang 36531, South Korea

**Keywords:** EpOME, *Frankliniella occidentalis*, hot pepper, nonstructural protein S (NSs), resistance-breaking, tomato spotted wilt virus (TSWV)

## Abstract

Tomato spotted wilt virus (TSWV) is a highly destructive plant pathogen transmitted by thrips, including *Frankliniella occidentalis*, in a circulative and propagative manner. To counter viral infections, thrips activate antiviral defences through C20 oxygenated polyunsaturated fatty acids (PUFAs), known as eicosanoids. However, at later stages of infection, C18 PUFAs, including epoxyoctadecamonoenoic acids (EpOMEs), modulate immune responses by preventing excessive and unnecessary activation. Our previous study demonstrated that TSWV elevates EpOME levels in thrips to suppress antiviral responses and enhance viral replication, with its nonstructural protein S (NSs) playing a key role in this process. In this study, we investigated the impact of NSs protein variation on vector immunity and virus–vector interactions. We assessed relative TSWV titres in thrips larvae and examined the role of eicosanoids, specifically 12,13-EpOME and PGE_2_, in regulating viral load and apoptosis. Our results revealed that 12,13-EpOME significantly increased viral titres, whereas PGE_2_ reduced the viral accumulation by promoting apoptosis in the vector insect. Phylogenetic analysis identified distinct *NSs* variations among TSWV isolates, with resistance-breaking (RB) and WT strains, which modulated differential infection patterns in thrips gut tissues, as visualized through fluorescence in situ hybridization. RB strains exhibited significantly higher viral titres, along with increased expression of EpOME biosynthetic gene (*Fo-CYP24*) and decreasing expression of EpOME degradation gene (*Fo-sEH2*). Apoptosis assays using the terminal deoxynucleotidyl transferase dUTP nick-end labelling assay further indicated that RB strains suppressed the gut epithelial cell death in thrips by antagonizing a process regulated by PGE_2_. Additionally, *in vivo* transient expression of the *NSs* gene in a nontarget insect, *Spodoptera exigua*, demonstrated the immunosuppressive effects by inducing EpOME level through upregulation of *Se-CYP* expression and downregulation of *Se-sEH* expression. Indeed, RB strains suppressed cellular immune responses more effectively than WT strains in *S. exigua*. These findings provide novel insight into the role of *NSs* genetic variation in TSWV transmission in the insect vector as well as in the host plants.

## Data Availability

Nonstructural protein S (NSs) sequences identified in this study have been deposited in GenBank with the following accession numbers: TSWV-A5 (PV472197), TSWV-A4 (PV472198), TSWV-SD2 (PV472199), TSWV-N1 (PV472200), TSWV-N4 (PV472201) and TSWV-SD8 (PV472202).

## Introduction

The tomato spotted wilt virus (TSWV), *Orthotospovirus tomatomaculae*, belongs to the family *Tospoviridae* and is one of the most economically significant viral pathogens affecting pepper (*Capsicum annuum*) worldwide [1,2]. The TSWV genome consists of three negative-sense or ambisense single-stranded RNA segments, in which the large RNA (~8,900 nt) encodes the RNA-dependent RNA polymerase, the medium RNA (~4,800 nt) encodes the nonstructural movement protein and the glycoprotein precursor, while the small RNA (~2,900 nt) encodes the RNA silencing suppressor (NSs) and the nucleocapsid N protein [3].

Breeding TSWV-resistant pepper cultivars has been a primary strategy for disease management. These cultivars predominantly rely on a single dominant resistance gene, *Tsw*, which provides resistance by triggering a hypersensitive response upon recognition of the NSs protein of TSWV [4]. However, the emergence of resistance-breaking (RB) TSWV strains has been reported in several countries, including Spain, Italy, Brazil and Korea [5,7]. In Korea, TSWV outbreaks were first recorded in 2003 [8], leading to the commercial release of *Tsw*-resistant pepper cultivars [9]. Unfortunately, in 2019, RB variants of TSWV emerged and presented a serious challenge to control the plant viral disease even in resistant host plants [6].

RB strains have been increasingly reported in regions where resistant tomato and pepper cultivars are widely cultivated. Surveys in California have shown that RB strains have risen in prevalence under intensive deployment of *Sw-5*-based resistance, with detections often outnumbering WT isolates in commercial fields [10]. Although resistant pepper cultivars remain effective in reducing overall incidence, RB isolates have been detected and can cause residual infection levels ranging from 6 to 17% in *Tsw*-resistant pepper fields [11]. Additionally, studies characterizing hot pepper resistance-breaking isolates reveal that RB strains can emerge and persist in pepper under selection pressure, though they may vary in competitiveness (e.g. high fitness costs or reversion events) [12].

The NSs protein has been identified as the key determinant for TSWV resistance breakdown [13]. Comparative sequence analysis of NSs proteins from RB and WT strains has revealed multiple amino acid substitutions, though no single conserved mutation has been consistently linked to resistance breaking across different geographic isolates [14]. For example, a single point mutation T104A, previously identified as an RB mutation [5], was absent in several RB isolates, further suggesting that TSWV adapts through a diverse range of mutations rather than a universal RB mechanism.

TSWV is transmitted by multiple thrips species, including *Frankliniella occidentalis*, in a persistent and propagative manner [15]. The thrips exhibit complex immune interactions with the virus [16,17]. As a propagative virus, TSWV must overcome multiple immune barriers within the thrips vector to establish infection and ensure transmission [18,20]. Despite this immune response, TSWV has evolved counter-defence strategies to suppress antiviral immunity in thrips. The NSs protein of TSWV functions as an RNA silencing suppressor, inhibiting the RNA interference (RNAi) machinery and facilitating viral replication in the vector [21,22]. In addition to RNAi suppression, thrips exhibit immune responses through the induction of autophagy and antimicrobial peptide production upon viral infection [23,24].

Among the central regulators of insect immune responses are eicosanoids, a diverse group of bioactive lipid mediators derived from polyunsaturated fatty acids (PUFAs) [25,26]. These molecules, including prostaglandins, leukotrienes and epoxyeicosatrienoic acids, play crucial roles in orchestrating both cellular and humoral immunity by modulating key signalling pathways and promoting pathogen clearance [27]. Upon infection, eicosanoid biosynthesis is rapidly upregulated through the activation of phospholipase A_2_ (PLA_2_), an enzyme that hydrolyses membrane phospholipids to release arachidonic acid (20 : 4 n-6) and other C20 PUFAs, which serve as precursors for enzymatic conversion into active eicosanoids [28]. This tightly regulated process ensures a rapid immune response to invading pathogens.

While most eicosanoids enhance immune defence, certain oxylipins, such as epoxyoctadecamonoenoic acids (EpOMEs), act as immunosuppressors [29]. EpOMEs, including vernolic acid (12,13-EpOME) and coronaric acid (9,10-EpOME), are synthesized by cytochrome P450 monooxygenases (CYPs) and subsequently degraded by soluble epoxide hydrolase (sEH) [30]. These immunosuppressive compounds have been shown to downregulate immune responses by inhibiting apoptosis and suppressing the expression of key immune genes, ultimately facilitating pathogen persistence within the host [31].

Our previous study provided insights into the role of the thrips immune system in facilitating TSWV transmission [32]. The study demonstrated that TSWV suppresses the antiviral immune response of *F. occidentalis* by upregulation and downregulation of *CYP* and *sEH* to elevate the levels of EpOMEs. These increases of EpOME interfere with apoptosis, reducing caspase gene expression and promoting viral replication. Furthermore, NSs was identified as a key virulence factor responsible for the elevation of EpOME levels in *F. occidentalis* [32].

In this study, we hypothesized that natural variation in the NSs protein of TSWV probably modulates the MYC/MAX transcriptional complex to alter EpOME metabolism and thereby influence antiviral responses in the insect vector. To test this, we compared RB and WT TSWV strains across plant, vector and non-vector systems, integrating infection assays, gene expression and metabolite analyses. Our results show that RB strains carry a distinct NSs polymorphism that enhances EpOME signalling, suppresses vector antiviral immunity and promotes more efficient virus transmission.

## Methods

### TSWV sampling and thrips insect rearing

During June to August 2024, a survey was conducted to detect TSWV in hot pepper plants (*C. annuum*) exhibiting symptoms such as yellowing, chlorotic ring spots and distortion. A total of 118 symptomatic samples were collected from greenhouses and fields in Andong, Korea. Samples were labelled and stored at −20℃ until the extraction of total RNA.

The laboratory population of *F. occidentalis* originated from hot pepper fields in Andong and was maintained under controlled conditions at 25±2 °C, with a photoperiod of 16:8 h (light/dark) and relative humidity of 60±5%. Germinated bean plants (*Phaseolus coccineus* L.) were provided for thrips feeding and oviposition. Newly laid eggs from adult colonies were transferred to breeding dishes (SPL Life Sciences, Pocheon, Korea), and fresh beans were supplied daily after egg hatch. Under these conditions, larvae passed through two instar stages (L1–L2), followed by development into prepupae, which are distinguishable from pupae by their visible wing pads.

### Chemicals

12,13-EpOME and prostaglandin E_2_ (PGE_2_) were obtained from Cayman (Ann Arbor, MI, USA). 12-(3-Adamantan-1-ylureido) dodecanoic acid (AUDA) was sourced from Sigma-Aldrich Korea (Seoul, Korea) and dissolved in DMSO for the preparation of testing solutions. 5-Bromo-2′-deoxyuridine (BrdU) and anti-BrdU antibody were procured from Abcam (Cambridge, UK). Terminal deoxynucleotidyl transferase, FITC-conjugated anti-mouse IgG antibody and DAPI were acquired from Thermo Fisher Scientific (Wilmington, DE, USA). BSA, DMSO and t-octylphenoxy-polyethoxyethanol (Triton X-100) were obtained from Sigma-Aldrich, Korea. PBS was formulated with 100 mM phosphoric acid containing 0.7% NaCl and adjusted to a pH of 7.4 using 1 M NaOH. An anticoagulant buffer (ACB, pH 4.5) was created using 186 mM NaCl, 17 mM Na_2_EDTA and 41 mM citric acid.

### TSWV identification

Total RNA was extracted from 0.5 g of leaf tissue collected from each sample using TRIzol reagent (Invitrogen, Carlsbad, CA, USA), according to the manufacturer’s instructions. RNA concentration and purity were measured using a NanoDrop spectrophotometer (Thermo Fisher Scientific). Subsequently, the extracted RNA was reverse-transcribed into cDNA using RT-premix containing random hexamer primers (Intron Biotechnology, Seoul, Korea), following the manufacturer’s protocol. One hundred nanograms of the synthesized cDNA was then used as a template for PCR amplification with specific primers targeting the TSWV *N* and TSWV *NSs* genes (Table S1, available in the online Supplementary Material). Real-time PCR conditions included an initial denaturation step at 94 ℃ for 5 min, followed by 35 cycles consisting of denaturation at 94 ℃ for 30 s, annealing at 52 ℃ (for TSWV *N*) or 54 ℃ (for TSWV *NSs*) for 30 s, and extension at 72 ℃ for 30 s. A final extension step was performed at 72 ℃ for 10 min.

### Amplification and sequence analysis of the complete TSWV *NSs* gene

To determine the nucleotide sequence of the full *NSs* gene from six TSWV isolates (Table S2), amplification was performed using specific primers (Table S1). All amplified fragments with the expected sizes (~ 1,400 bp) were extracted and purified from agarose gels using a gel extraction kit (GeneAll Biotechnology, Seoul, Korea) according to the manufacturer’s instructions, and sequenced by Macrogen Inc. (Seoul, Korea) in both directions using primer walking. Nucleotide sequences of amplified fragments were analysed by online blastn software (http://blast.ncbi.nlm.nih.gov/Blast.cgi), and the NSs ORF was predicted using the online ORF Finder software (www.ncbi.nlm.nih.gov/projects/gorf/) to identify start and stop codons.

Relationships among the six selected TSWV isolates (TSWV-A4, TSWV-A5, TSWV-SD2, TSWV-N1, TSWV-N4 and TSWV-SD8) were determined by neighbour-joining phylogenetic analysis based on the deduced amino acid sequences of the NSs protein using 1,000 bootstrap replicates in mega 11 software [33]. The amino acid sequences of the NSs ORF from our six TSWV isolates were multiple aligned with publicly available sequences of RB and WT isolates (Table S3) using clustal w implemented in mega 11 software.

### TSWV inoculation and disease severity indexing

To discriminate RB and WT strains of TSWV, six selected TSWV isolates were inoculated on susceptible (*C. annuum* cv. Chungyang, Jungang Farm, Okcheon, Korea) and resistant (*C. annuum* cv. Color-Jjang, Nongwoo Bio, Suwon, Korea) hot pepper cultivars in early stages of growth, using carborundum as an abrasive. The inocula were prepared by homogenizing TSWV-infected hot pepper leaf tissues in 0.1 M phosphate buffer (pH 7, 1:10, w/v), containing 0.2% sodium diethyldithiocarbamate and 1% polyvinylpyrrolidone as virus stabilizers. The inoculated plants were kept in a phytotron at 28 °C with a 16:8 h light/dark photoperiod and observed for symptom development for 14 days. TSWV titre of all inoculated plants was measured by qualitative PCR (qPCR) at 14 days post-inoculation.

The disease severity of the selected TSWV isolates on inoculated hot peppers was assessed visually, recording symptoms 14 days post-inoculation using a disease indexing score from 1 (no symptoms) to 5 (severe necrosis and stunting), as described by Kim *et al*. [34]. Strains were classified as RB or WT a priori using both phenotypic and molecular criteria. RB strains were defined as those producing clear systemic symptoms in resistant plants with a mean disease severity index ≥3.0 (on a 1–5 scale). TSWV titre was measured by real-time qPCR using the TSWV-*N* gene, normalized against the reference gene *β-actin* in both susceptible and resistant hot peppers (described below). Each isolate was tested in three independent biological replicates, each with three technical replicates.

### Bioinformatics to predict transcription factors (MYC and MIX) in *F. occidentalis*

The MYC protein sequence of *Arabidopsis thaliana* (AtMYC2), characterized by a basic helix-loop-helix (bHLH) leucine zipper domain (Table S4), was used as a query sequence to identify orthologous transcription factors in the transcriptomes of *F. occidentalis*, *Bombyx mori*, *Drosophila melanogaster*, *Apis mellifera* and *Spodoptera exigua*. The AtMYC2 amino acid sequence was compared against insect transcriptome databases available in NCBI GenBank (https://www.ncbi.nlm.nih.gov/) using protein blast. Candidate sequences identified from these searches were aligned using Clustal W in mega 11 software [33], resulting in the identification of insect *MYC* and *MAX* genes (Table S4). A phylogenetic tree based on these amino acid sequences was constructed using the neighbour-joining method with 1000 Bootstrap replicates in mega 11 to estimate node support. Conserved protein domains were predicted by querying the NCBI Conserved Domain Database (https://www.ncbi.nlm.nih.gov/cdd).

### TSWV infection in thrips

To prepare viral suspensions, approximately 100 mg of symptomatic leaf tissue from each of the six selected TSWV isolates was homogenized in 1 ml of filter-sterilized (0.22 µm pore size) PBS. After centrifugation at 14,000×***g*** for 5 min, the supernatant was collected and used as the viral inoculum. Prior to virus exposure, first (L1) and second instar (L2) larvae were starved for 1 h. Larvae were subsequently exposed to TSWV using a feeding-based inoculation method: germinated bean seed kernels were immersed in 1 ml of viral suspension for 5 min and then air-dried for 10 min under sterile conditions. The treated kernels were placed in small breeding petri dishes (100×40 mm, SPL Life Sciences), and larvae were allowed to feed on these kernels for 12 h. Afterwards, virus-treated kernels were replaced with fresh, untreated kernels.

### Insect sample preparation to quantify EpOMEs

First instar (L1) larvae of *F. occidentalis* were used for the EpOME extraction. Approximately 1,500 larvae per sample were collected after a 24 - feeding period on a TSWV-infected diet and rinsed three times with chilled PBS. This procedure was independently replicated three times for each treatment. EpOME extraction was carried out following the method described by Hrithik *et al*. [35]. Briefly, samples were homogenized using an ultrasonicator (Bandelin Sonoplus, Berlin, Germany) at 80% power for three cycles of 1 min each in PBS. The sample pH was then adjusted to 4 using 1 N HCl. Subsequently, each sample was mixed with 1 ml of ethyl acetate to facilitate phase separation, and the organic (upper) phase was collected. The aqueous (lower) phase was further extracted twice with ethyl acetate. The combined ethyl acetate extracts were concentrated to ~500 µl under a gentle stream of nitrogen gas and then applied onto a small silicic acid column (2×90 mm; containing 30 mg silicic acid, Type 60A, 100–200 mesh, Sigma-Aldrich Korea). Extracts were sequentially eluted using 250 µl volumes of solvents with increasing polarity, starting with 100% ethyl acetate, followed by ethyl acetate–acetonitrile (1  1, v/v) and acetonitrile–methanol (1:1, v/v), and concluding with 100% methanol. The ethyl acetate fraction was then used for EpOME quantification. Each treatment was replicated three times with independently prepared samples.

### LC-MS/MS analyses

Liquid chromatography-tandem mass spectrometer (LC-MS/MS) analysis was carried out using a QTrap 4500 (AB Sciex, Framingham, MA, USA) equipped with an auto-sampler, a binary pump and a column oven. The analytical column was a C18 column (2.1×150 mm, 2.7 µm, Osaka Soda, Osaka, Japan) maintained at 40 °C. The mobile phases consisted of 0.1% formic acid in water and 0.1% formic acid in acetonitrile. The gradient programme started at 30% B at 0 min, maintained 30% B at 2 min, increased to 65% B by 12 min, further increased to 95% B by 12.5 min, remained at 95% B up to 25 min, then reduced to 30% B at 28 min and held at 30% B until 30 min. The flow rate was set at 0.40 ml min^−1^. The auto-sampler temperature was set at 5 ℃, and the injection volume was 10 µl. The LC-MS/MS was equipped with an electrospray ionization source operating in negative ion mode. After optimization, the source parameters were set as follows: temperature at 600 ℃, curtain gas flow rate at 32 l min^−1^, ion gas flow rate at 60 l min^−1^ and spray voltage adjusted at 4,000 V. Analytical assessments were performed using multiple reaction monitoring detection mode with nitrogen as the collision gas. Mass View 1.1 software (AB Sciex) was used for peak detection, integration and quantitative analysis.

### RNA extraction, cDNA synthesis, real-time PCR and real-time qPCR

Total RNA was extracted from ~50 individuals of *F. occidentalis* at various developmental stages (larvae, pupae, adult males and adult females) as described above. RNA concentrations were quantified using a NanoDrop spectrophotometer (Thermo Fisher Scientific), and cDNA synthesis was performed using RT-premix containing oligo-dT primers (Intron Biotechnology), following the manufacturer’s protocol. Gene expression analysis was conducted using a StepOnePlus Real-Time PCR System (Applied Biosystems, Singapore) and Power SYBR Green PCR Master Mix (Toyobo, Osaka, Japan), following the manufacturer’s guidelines. Each real-time qPCR reaction (20 µl total volume) included 10 µl of 2×Power SYBR Green PCR Master Mix, 100 ng of cDNA template and 10 pmol of each gene-specific primer (Table S1). PCR cycling conditions consisted of an initial denaturation at 95 ℃ for 10 min, followed by 40 cycles of 98 ℃ for 20 s, 52 ℃ for 30 s and 72 ℃ for 1 min. The elongation factor 1 (*EF1*) and *β-actin* genes were used for reference to normalize target gene expression levels or TSWV titre in *F. occidentalis* and hot peppers, respectively (Table S1). Melting curve analyses were performed to confirm the specificity of PCR products. Each treatment was independently replicated three times, and relative gene expression levels were calculated using the comparative CT method [36].

### dsRNA preparation and RNAi

dsRNAs were synthesized using a MEGAscript RNAi kit (Ambion, Austin, TX, USA), strictly following the manufacturer’s instructions. Initially, genes were amplified via PCR using gene-specific primers that incorporated a T7 RNA polymerase promoter sequence at the 5′ end (Table S1). This PCR product served as the template for dsRNA synthesis. Both sense and antisense RNA strands were generated using T7 RNA polymerase at 37 °C over a period of 4 h. The dsRNA was purified and mixed with the transfection agent Metafectene PRO (Biontex, Planegg, Germany) at a 1 : 1 (v/v) ratio and incubated at 25 °C for 30 min to facilitate liposome formation. Subsequently, the dsRNA–liposome complex was administered to larvae using a feeding protocol in which beans were immersed in a 500 µg ml^−1^ dsRNA solution for 20 min. The efficiency of RNAi was assessed at intervals of 0 (just after injection), 6, 12, 24 and 48 h post-treatment through real-time qPCR, as previously described. Each experimental condition was replicated three times. A control dsRNA was synthesized from a 520-bp segment of the viral gene *CpBV302* [37]. dsRNA against *Fo-sEH2*, *Fo-CYP24*, *TSWV-NSs*, *TSWV-N* and *TSWV-NSm* was described before [32].

### Fluorescence in situ hybridization assay

After a 24-h feeding period on a TSWV-infected diet, the first instar (L1) larvae were dissected to isolate their guts, which were transferred onto sterilized glass slides and fixed in 4% paraformaldehyde for 1 h at room temperature (RT). Following fixation, samples were rinsed in 1×PBS and permeabilized with 1% Triton X-100 in PBS for 2 h at RT. After an additional PBS wash, the guts were rinsed with 2×saline sodium citrate (SSC) and incubated at 42 ℃ in 25 µl of pre-hybridization buffer (2 µl yeast tRNA, 2 µl 20×SSC, 4 µl dextran sulphate, 2.5 µl 10% SDS and 14.5 µl deionized H₂O) for 1 h in a dark and humid environment. The pre-hybridization buffer was then replaced with a hybridization buffer consisting of 5 µl deionized formamide, 1 µl fluorescein-labelled oligonucleotide probe and 19 µl of the initial pre-hybridization mixture. Fluorescently labelled DNA oligonucleotide probes targeting the *TSWV N* gene [29] were synthesized with fluorescein amidite at their 5′ ends and purified via high-performance liquid chromatography (Bioneer, Daejeon, Korea). After covering slides with RNase-free coverslips, samples were incubated overnight (18 h) at 42 ℃ in a humid chamber. Post-hybridization washing steps included two washes in 4×SSC (10 min each), followed by incubation in 4×SSC with 1% Triton X-100 for 5 min at RT, and three additional washes in 4×SSC. Subsequently, samples were incubated at 37 ℃ for 30 min in the dark with 1% anti-rabbit-FITC-conjugated antibody (Thermo Fisher Scientific) diluted in PBS. The guts were washed twice with 4×SSC (10 min each), followed by a final wash with 3×SSC, and allowed to air-dry. A drop of 50% glycerol was applied, samples were incubated for 15 min at RT, covered with a coverslip and examined under a fluorescence microscope (DM2500, Leica, Wetzlar, Germany) at ×200 magnification.

### Terminal deoxynucleotidyl transferase dUTP nick-end labelling assay

Terminal deoxynucleotidyl transferase dUTP nick-end labelling (TUNEL) assays were performed using an *in situ* Cell Death Detection Kit (Abcam). Twelve hours post-TSWV infection, guts from L1 larvae were carefully dissected in PBS and placed on coverslips (22×22 mm). Tissues were incubated with 10 µM BrdU and terminal transferase for 1.5 h. Following incubation, tissues were fixed with 4% paraformaldehyde at RT for 1 h, rinsed with PBS and permeabilized using 0.3% Triton X-100 in PBS for 2 h. Samples were then blocked with 5% BSA in PBS for 1 h. Subsequently, the guts were incubated with anti-BrdU antibody (1:15 dilution in blocking solution) for 1 h at RT. After three washes in PBS to remove unbound antibodies, the samples were incubated for 1 h at RT with FITC-conjugated anti-mouse IgG antibody (1:300 dilution in blocking solution). Following three additional PBS washes, samples were stained with DAPI (1:1000 dilution in blocking solution) for 5 min at RT. After a final PBS rinse, samples were mounted onto glass slides using 10 µl of glycerol–PBS solution. Slides were observed under a fluorescence microscope (DFC450C, Leica) using FITC mode. Each treatment was independently replicated three times.

### Preparation of recombinant pIB-NSs vector

The recombinant pIB-NSs vector was constructed for six RB and WT selected *NSs* genes according to a previous study [32]. Briefly, the PCR product containing the *NSs* ORF (1,221 bp) was cloned into the pIB vector (pIB/V5-His TOPO TA Expression Kit, Invitrogen) for *in vivo* transient expression (IVTE). Following confirmation of the insertion direction by PCR, the recombinant vector was sequenced to verify that the ORF was correctly positioned in the reading frame under the baculoviral immediate early promoter (OplE2).

### IVTE of NSs in a non-host, *S. exigua*

Larvae were obtained from a laboratory strain of *S. exigua* reared by the method of Goh *et al*. [38]. IVTE followed the method described by Hepat and Kim [39]. Briefly, fourth instar larvae of *S. exigua* were injected with a 1 µl mixture of pIB-NSs vector (200 ng) and a transfection reagent (=Metafectene Pro) at 1 : 1 ratio using a Hamilton microsyringe. To analyse the expression, RNA was extracted from the test larvae at 0, 6, 12, 24 and 48 h post-injection (pi), and subsequent qPCR was performed to quantitatively analyse the gene expression as described above.

### Quantification of cAMP and Ca^2+^ signals

cAMP measurement was performed using a cyclic AMP ELISA Kit (Cayman Chemical, Ann Arbor, MI, USA). Fifth instar larvae of *S. exigua* were injected with six pIB-NSs vectors (200 ng) as described above. At 24 h pi, hemolymph (50 µl) from the test larvae was mixed with 148 µl of ACB, in which 1 µl of PGE_2_ (10^−7^ M) was added. As a control, an empty pIB vector (200 ng) was injected into L5 larvae. The mixture was centrifuged at 1,000×***g*** for 10 min at 4 ℃. The cell pellet was lysed in 100 µl of 0.1 M HCl for 20 min at RT, and the supernatant was collected after a second centrifugation at 1,000×***g*** for 10 min at 4 ℃. The supernatant was acetylated by adding 50 µl of 0.4 M KOH and acetic anhydride provided with the kit. After acetylation, 50 µl of each sample or standard was added to the specified well followed by the sequential addition of 50 µl of cAMP AChE Tracer and 50 µl of cAMP EIA antiserum. The plate was incubated overnight at 4 ℃. After washing five times with 200 µl of washing buffer, 200 µl of Ellman’s reagent was added and incubated at RT in the dark until colour development. To measure the cAMP in *F. occidentalis*, L1 larvae were fed six recombinant pIB-NSs vectors (200 µg ml^−1^) as described above. At 24 h post-treatment, treated larvae were homogenized in PBS and used to measure the cAMP level as described above. Absorbance was read at 405 nm. The cAMP concentration was calculated by plotting an equation using known standard concentrations, and the unknown concentration was determined based on the observed value at 405 nm.

To assess changes in Ca^2+^ signals in haemocytes in response to *NSs* gene expression, pIB-NSs vectors (200 ng) were injected into individual L5 larvae of *S. exigua*. For control, an empty pIB vector (200 ng) was injected. After 24 h, the test larvae were injected with 2 µl of Fura-8AM (1 mM) along with PGE_2_ (10^−7^ M). One hour pi, hemolymph was collected and fixed on a slide glass using 2.5% paraformaldehyde. For *F. occidentalis*, L1 larvae were fed with pIB-NSs recombinant vectors (200 µg ml^−1^). After 24 h, larvae were fed with Fura-8AM (1 mM) along with PGE_2_ (10^−7^ M). Twelve hours post-treatment, guts from L1 larvae were carefully dissected in PBS and fixed on a slide glass using 2.5% paraformaldehyde. Fura-positive cells were observed under a fluorescence microscope at 200× magnification. Fluorescence intensity was analysed using ImageJ software (https://imagej.nih.gov/ij).

### Haemocyte-spreading behaviour

To analyse the haemocyte-spreading behaviour in response to NSs expression, vectors were administered as described above to fifth instar larvae of *S. exigua*. After 24 h, total haemolymph (250 µl) was obtained and combined with 750 µl of ACB. The haemocyte suspension was incubated on ice for 30 min. After centrifugation at 800×***g*** for 5 min, 700 µl of supernatant was discarded, and the cell pellet was gently resuspended in 700 µl of TC-100 insect tissue culture medium (Welgene, Gyeongsan, Korea). A 19 µl aliquot of the haemocyte suspension was incubated with 1 µl of PGE_2_ (10^−7^ M) on a glass coverslip for 1 h at RT. As a control, an empty pIB-NSs vector was injected into the larvae. Cells were then fixed with 4% paraformaldehyde for 10 min at RT. After washing three times with PBS, cells were permeabilized with 0.2% Triton X-100 in PBS for 2 min at RT, washed once with PBS and blocked with 10% BSA in PBS for 10 min at RT. After another PBS wash, cells were incubated with FITC-tagged phalloidin in PBS for 1 h at RT. Following three washes, cells were stained with DAPI (1 mg ml^−1^) in PBS for nuclear staining. After two final PBS washes, cells were observed under a fluorescence microscope (DM2500, Leica) at 200× magnification. Haemocyte spreading was assessed based on the extension of F-actin beyond the original cell boundary. For each behavioural assay, 100 randomly selected cells were analysed. Each treatment was replicated three times using independently prepared samples.

### Nodulation assay

At 24 h after IVTE treatment, *Escherichia coli* (2×10^4^ cells per larva) was injected into individual larva of *S. exigua* and incubated for 8 h at RT. Then, the test larvae were dissected to expose the hemocoel, and melanized nodules were counted under a stereoscopic microscope (Stemi SV11, Zeiss, Jena, Germany) at 50× magnification. Each treatment was performed in triplicate.

### Statistical analysis

All the continuous variable data were analysed by ANOVA with the PROC GLM procedure in the SAS program [40]. Percentage data were arcine-transformed for normalization. Means were compared using the least significant difference (LSD) test at type I error=0.05. All the graphs in this study were prepared using GraphPad Prism v. 8.0.1 (Boston, MA, USA).

## Results

### Two oxylipins modulate TSWV titres in *F. occidentalis*

**Fig. 1.**
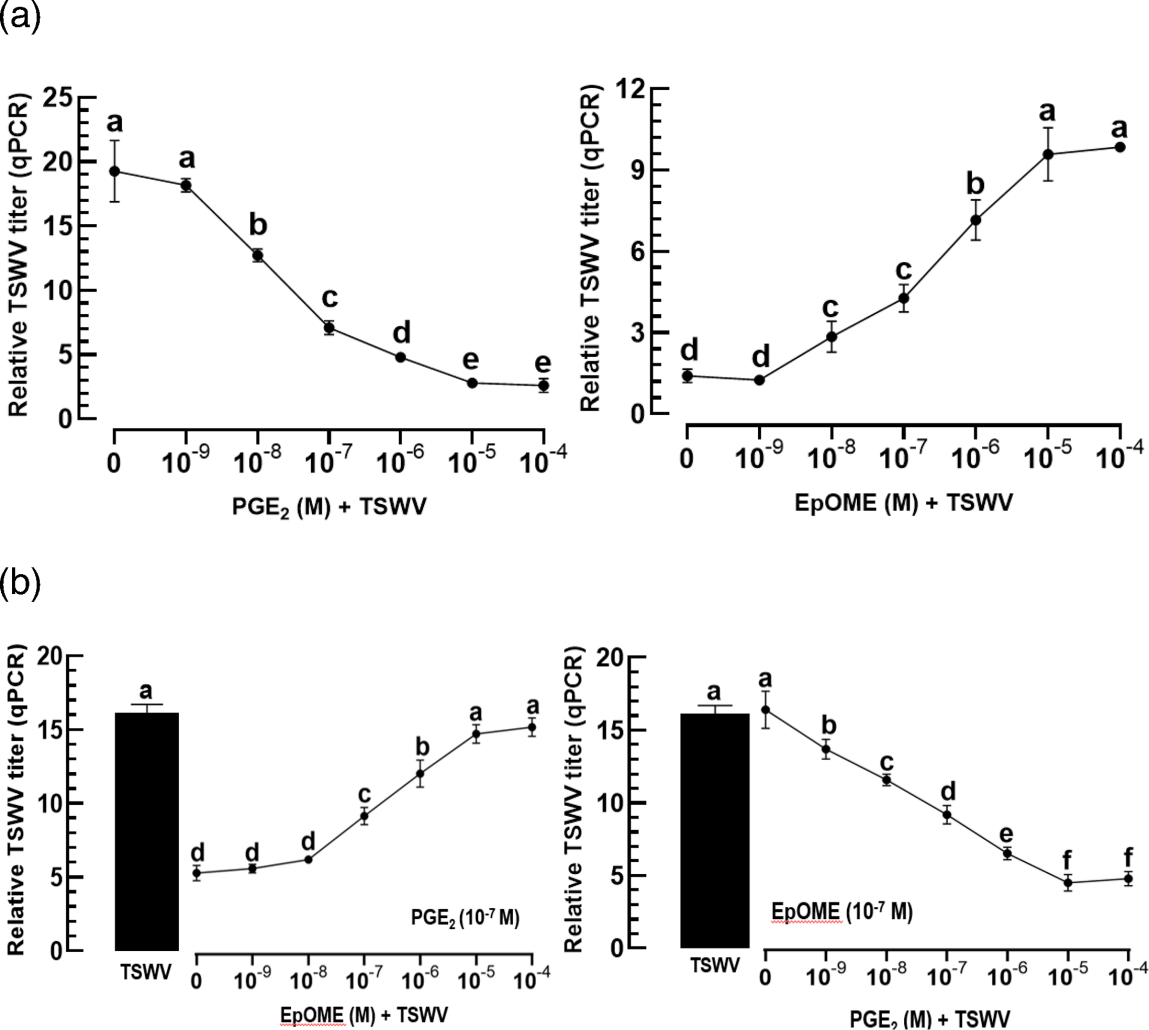
Opposite modulations of PGE_2_ and 12,13-EpOME on TSWV titres of the viruliferous larvae of *F. occidentalis*. (**a**) Dose-dependent modulations of the two oxylipins. The first instar larvae were fed with TSWV and different doses of the oxylipins for 12 h. After 24 h post-treatment, the test larvae were used for analysis of the viral titre using real-time qPCR. (**b**) Antagonistic effects of the two oxylipins on the modulation of the viral titres. The first instar larvae were fed with TSWV and different doses of one oxylipin under a constant dose of the other oxylipin for 12 h. After 24 h post-treatment, the test larvae were used for analysis of the viral titre using real-time qPCR. ‘TSWV’ stands for the treatment without any addition of the oxylipins to the viral infection. Each treatment consisted of ~50 insects and was replicated three times. Different letters above standard error bars indicate significant differences among means at type I error=0.05 (LSD test).

PGE_2_, a C20 oxylipin, mediates insect immune responses, while 12,13-EpOME, a C18 oxylipin, suppresses these responses [26]. The addition of PGE_2_ to TSWV infection reduced the viral titre in thrips in a dose-dependent manner (Fig. 1a). In contrast, the addition of EpOME to TSWV infection increased the viral titres in a concentration-dependent manner. To clarify the antagonistic influence of the two oxylipins, they were applied together to the viral infection to test their interaction in varying molar ratios: increasing PGE_2_ concentration under a constant EpOME level or vice versa (Fig. 1b). As expected, viral titres changed in the direction of the increasing concentration of the opposite oxylipin in a competitive manner.

**Fig. 2.**
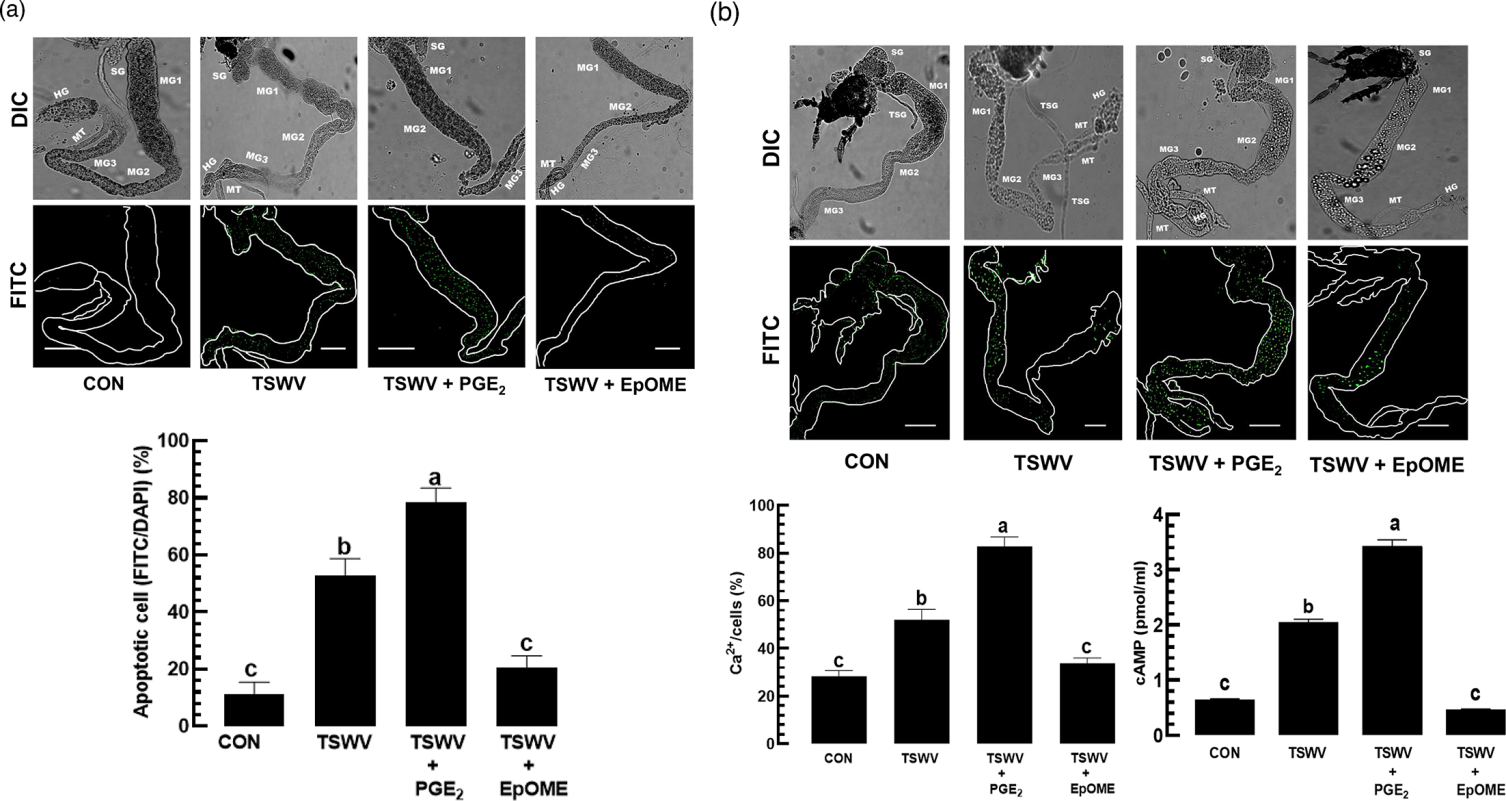
Antagonistic effects of PGE_2_ and 12,13-EpOME on the antiviral response of *F. occidentalis*. (**a**) Opposite modulations of apoptosis in the gut epithelium of *F. occidentalis* by the two oxylipins. The first instar larvae were fed with TSWV and different doses of the oxylipins for 12 h: PGE_2_ at 10^−7^ M and EpOME at 0.1 µg ml^−1^. After 12 h post-treatment, the test larvae were used for analysis of apoptosis by the TUNEL assay. An anti-mouse IgG antibody specific to BrdU confirmed a positive TUNEL response under a fluorescent microscope (DM2500, Leica, Wetzlar, Germany). The whole intestine and the apoptotic cells were observed by differential interference contrast (DIC) and fluorescein isocyanate (FITC) modes, respectively. Both FITC and DAPI signals were quantified in 100 randomly selected cells across three replicates. Scale bars represent 10 µm. ‘TSWV’ stands for the treatment without any addition of the oxylipins to the viral infection. Control (CON) represents naïve larvae without any viral or oxylipin treatments. (**b**) Opposite regulations of Ca^2+^ and cAMP in the gut epithelium by the two oxylipins. The entire gut and Ca^2+^ signal were observed by DIC and FITC modes, respectively, under four different treatments. FITC-positive cells were counted among randomly selected 100 cells stained by DAPI. Quantification of cAMP used a cyclic AMP ELISA kit (Cayman Chemical, Ann Arbor, MI, USA). Each treatment was replicated three times. Different letters above standard error bars indicate significant differences among means at type I error=0.05 (LSD test). Scale bar represents 0.1 mm.

To further analyse the antagonistic influence of the two oxylipins on viral titre, apoptosis in the gut epithelium was assessed (Fig. 2a). In response to TSWV infection, gut epithelia of *F. occidentalis* larvae exhibited apoptosis as shown by the TUNEL assay, in which PGE_2_ upregulated apoptosis while 12,13-EpOME suppressed it.

PGE_2_ binds to a specific membrane receptor and upregulates cAMP and subsequently calcium ion levels in insect haemocytes [7,41, 42]. TSWV infection increased cAMP and calcium ion levels in *F. occidentalis* (Fig. 2b). Addition of PGE_2_ to the viral infection further increased these secondary messengers to promote apoptosis against TSWV infection, while addition of EpOME decreased their levels.

### TSWV upregulates EpOME biosynthesis via *NSs*

**Fig. 3.**
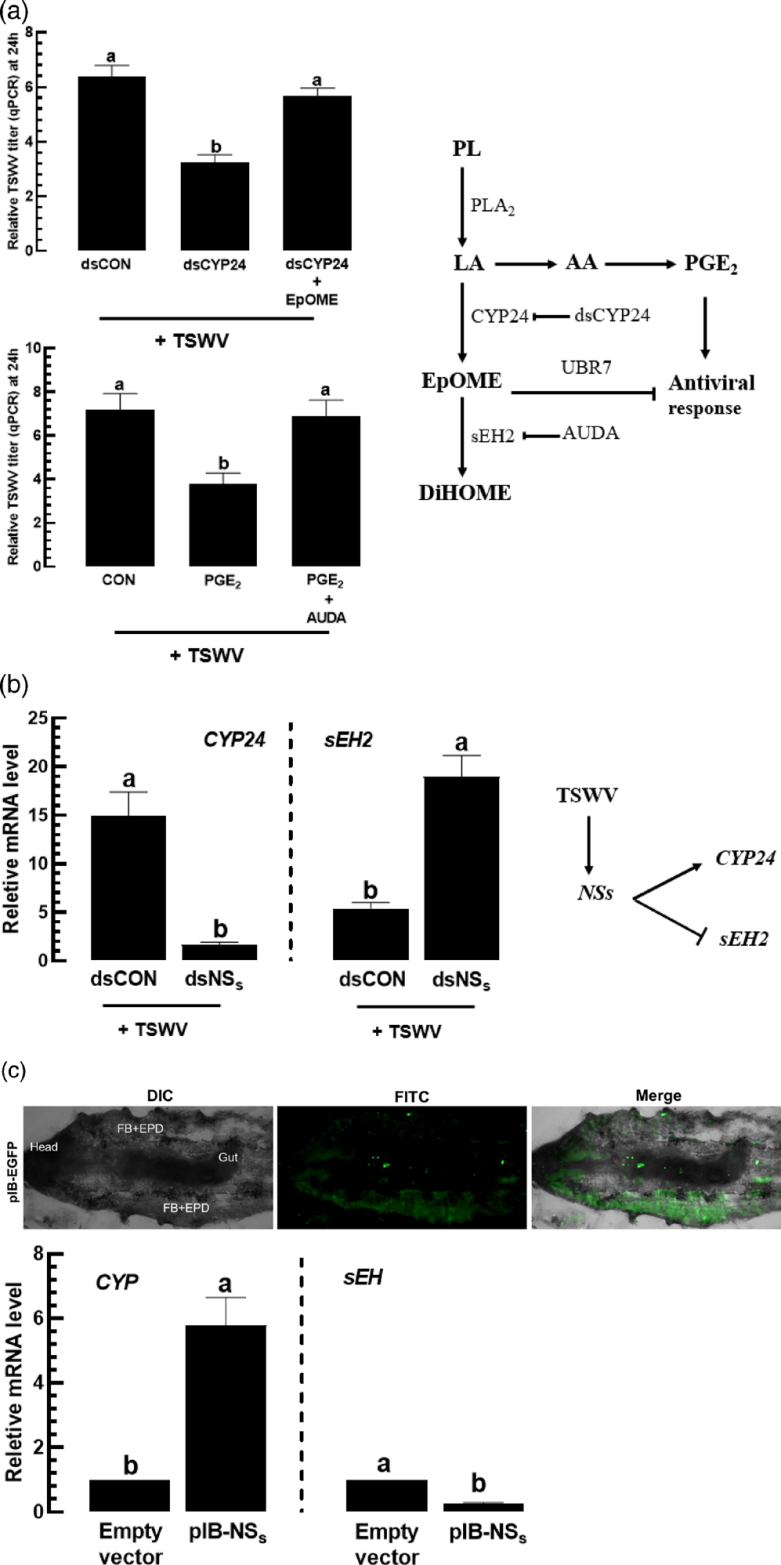
Upregulation of EpOME biosynthetic machinery by NSs expression of TSWV in *F. occidentalis*. (**a**) Increase in TSWV titre by EpOME. A dsRNA (dsCYP24) specific to *Fo-CYP24* was fed to L1 larvae at 500 µg ml^−1^. After 12 h post-dsRNA treatment, 12,13-EpOME at 0.1 µg ml^−1^ was fed to the larvae along with TSWV. A non-target gene, *CpBV302*, served as a control dsRNA (dsCON) along with TSWV infection. PGE_2_ at 10^−7^ M with or without AUDA (10 ppm) was fed to the larvae along with TSWV. The viral titres in the thrips were assessed 24 h post-treatment using real-time qPCR. The diagram explains the EpOME biosynthesis and degradation from phospholipid (PL). Linoleic acid (LA) is released by the catalytic activity of phospholipase A_2_ (PLA_2_) and oxygenated into EpOME by a cytochrome P450 oxygenase (CYP24). EpOME is then degraded into DiHOME by a soluble epoxide hydrolase (sEH). LA is changed into PGE_2_ via arachidonic acid (AA). AUDA is a specific inhibitor of sEH. An E3 ubiquitin-protein ligase (UBR7) is a downstream enzyme of EpOME to inhibit antiviral response. (**b**) Modulation of *Fo-CYP24* and *Fo-sEH2* expressions by *NSs* expression. A dsRNA (dsNSs) specific to *NSs* was fed to L1 larvae at 500 µg ml^−1^. After 12 h post-dsRNA treatment, the larvae were fed with TSWV. After 24 h, *Fo-CYP24* and *Fo-sEH2* were assessed in their expression levels using real-time qPCR. (**c**) Effect of *NSs* expression on EpOME synthetic (=*Se* CYP) and degrading (=*Se* sEH) genes in *S. exigua* larvae. The viral gene, *NSs*, was expressed in the larvae using IVTE by injecting the NSs-recombinant expression vector (200 ng/larva) into L5 larvae. Photos demonstrated the *EGFP* expression in different *S. exigua* tissues using this IVTE: fat body (FB), epidermis (EPD) and gut. After the *NSs* expression using the IVTE technique, *Se-CYP* and *Se-sEH* expressions were analysed by real-time qPCR at 24 h post-IVTE. Different letters above standard error bars indicate significant differences among means at type I error=0.05 (LSD test).

EpOME is synthesized from linoleic acid by epoxidation catalysed by a specific cytochrome P450 oxygenase (*CYP24*) and degraded by a soluble epoxidase (*sEH2*) in *F. occidentalis* (Fig. 3a) [32]. RNAi targeting *CYP24* expression significantly suppressed TSWV titre, and addition of EpOME restored the viral titre, supporting the role of EpOME in promoting viral replication. This was further supported by the effect of AUDA (=a specific inhibitor of sEH enzyme activity) on viral titre regulation, which antagonized the antiviral effect of PGE_2_, presumably by elevating the endogenous EpOME level.

TSWV may control the EpOME level in *F. occidentalis* by regulating the expression of *CYP24* and *sEH2*. Notably, NSs plays a crucial role in the upregulation of EpOME in *F. occidentalis* [31]. In fact, RNAi targeting *NSs* significantly reduced *CYP24* expression while increasing *sEH2* expression level (Fig. 3b). This regulation of EpOME metabolism by NSs was tested in a non-target insect, *S. exigua*, using *in vivo* transient expression (Fig. 3c). Heterologous expression was accomplished by a hemocoelic injection using a pIB expression vector under a baculovirus-derived immediate early promoter. *EGFP* expression confirmed successful transgene delivery in gut, fat body and haemocytes (upper panels in Fig. 3c). The haemocoelic injection of the *NSs*-recombinant vector significantly increased *CYP* expression and decreased *sEH* expression in *S. exigua* (lower panels in Fig. 3c), where *CYP* and *sEH* are directly involved in EpOME biosynthesis and degradation, respectively, in the insect [26,30].

### TSWV modulates transcription factors, MYC/MAX, to increase the EpOME level

**Fig. 4.**
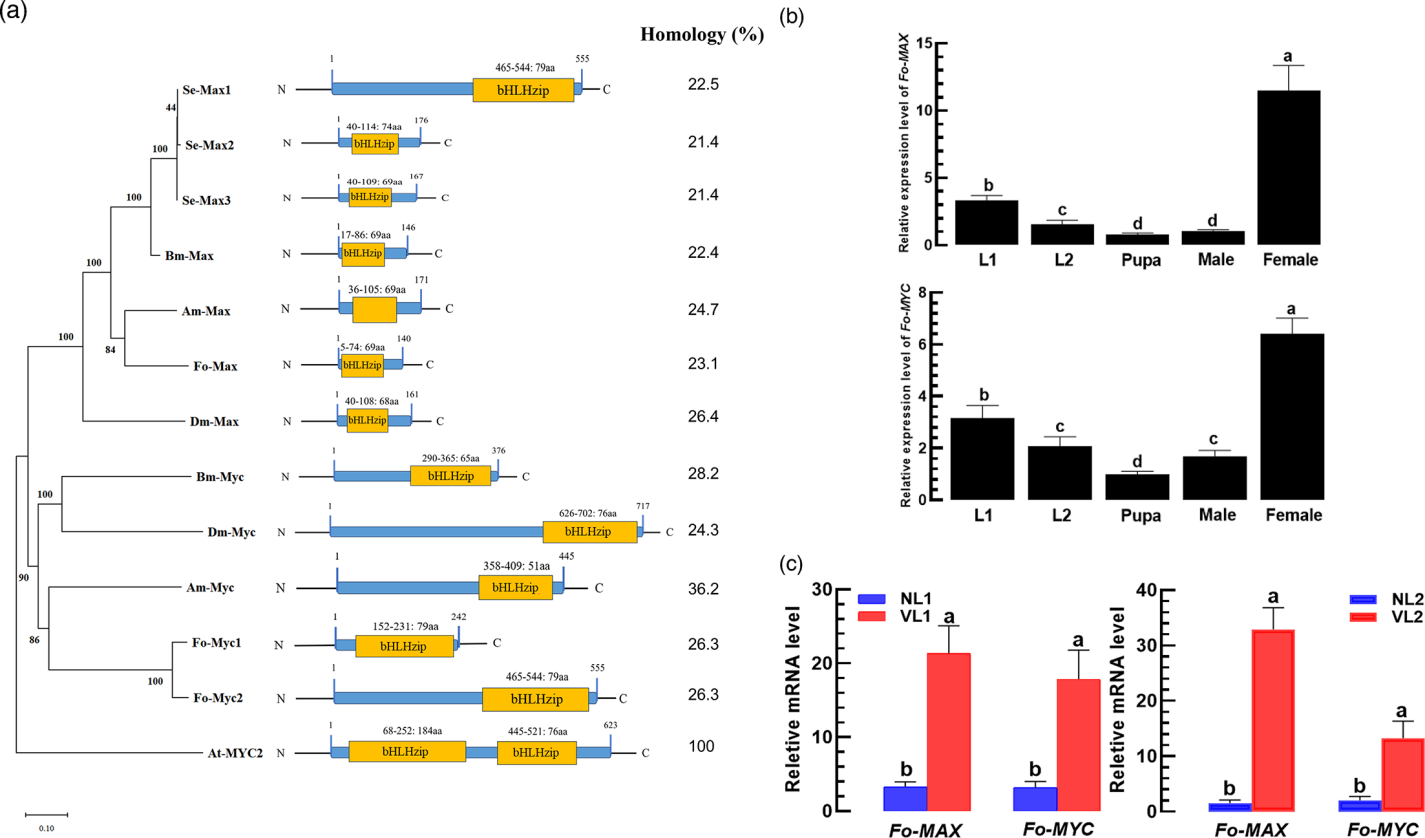
Prediction of *A. thaliana* MYC2 (*At-MYC2*) orthologues in *F. occidentalis*. (**a**) Genetic relatedness of MYC orthologues in insects based on their amino acid sequences (see Table S4 for their GenBank accession numbers). A phylogenetic tree was constructed by neighbour-joining clustering using mega 11, in which bootstrapping values were obtained from 1,000 repetitions to support the branching and clustering. ‘bHLHzip’ represents a basic helix-loop-helix zipper domain. ‘Homology’ represents the sequence homology (%) with the sequence of *At-MYC2*. (**b**) Expression profiles of two MYC orthologues (*Fo-MAX* and *Fo-MYC2*) in different developmental stages of *F. occidentalis*, including the first and second larvae (L1 and L2). (**c**) Upregulation of *Fo-MAX* and *Fo-MYC2* expressions upon TSWV infection of L1 (VL1) and L2 (VL2) larvae compared to non-viruliferous larvae (NL1 and NL2). Different letters above standard error bars indicate significant differences among means at type I error=0.05 (LSD test).

In host plants, NSs functionally bind to the transcription factor MYC to interfere with jasmonic acid in order to suppress insect resistance against the viral vector [43]. To predict the insect MYC orthologues, we used the *A. thaliana MYC2* gene (*At-MYC2*) to query insect genomes, including that of *F. occidentalis* (Fig. 4a). Two types of transcription factors showed relatively high homologies (21.4 –36.2%) with *At-MYC2*, with 26.3 and 23.1% for *Fo-MYC1/2* and *Fo-MAX* of *F. occidentalis*, respectively. All MAX and MYC proteins possess a basic helix-loop-helix-zipper (bHLH-ZIP) domain similar to At-MYC2. However, the *MAX* and *MYC* genes were clustered separately in insects. *Fo-MYC* and *Fo-MAX* were expressed at all developmental stages of *F. occidentalis*, with the highest expression observed in adult females (Fig. 4b). Upon TSWV infection, their expressions were highly induced, especially at the L1 and L2 larval stages (Fig. 4c).

**Fig. 5.**
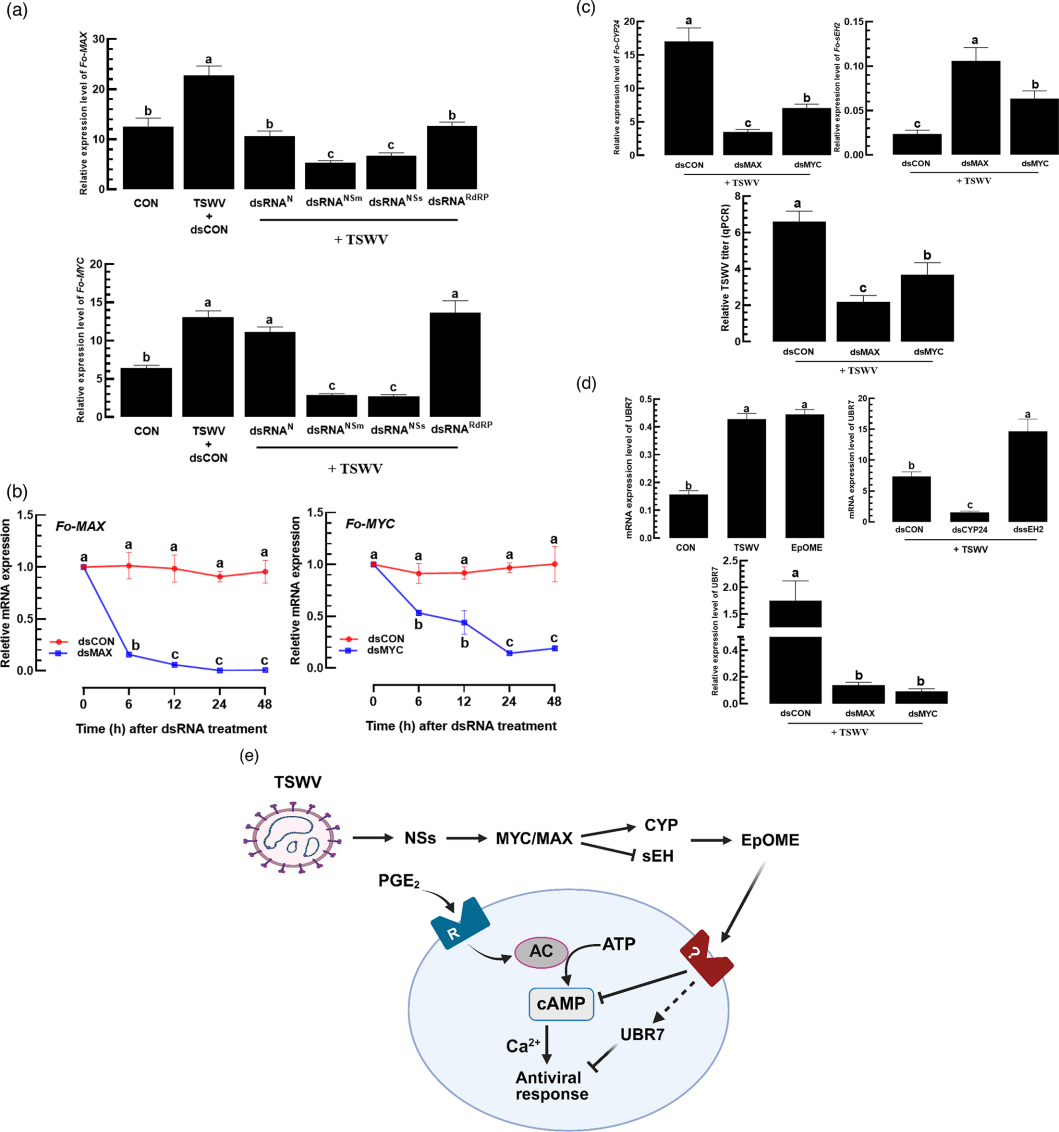
Role of two MYC orthologues of *F. occidentalis* in upregulating EpOME signal upon TSWV infection. (**a**) Influence of individual RNAi treatments specific to each TSWV gene (dsRNA^N^, dsRNA^NSm^, dsRNA^NSs^ and dsRNA^RdRP^) on expression of *Fo-MAX* and *Fo-MYC2*. Each dsRNA was fed to L1 larvae at 500 µg ml^−1^. After 12 h, the L1 larvae were infected with TSWV. The expression levels of *Fo-MAX* and *Fo-MYC2* were assessed at 24 h after the viral infection. A non-target gene, *CpBV302*, was used to prepare the control dsRNA (dsCON). Control (CON) treatment used non-viruliferous L1 larvae. (**b**) RNAi effect on *Fo-MAX* and *Fo-MYC2* expression levels by feeding individual gene-specific dsRNAs (dsMAX to *Fo-MAX* and dsMYC to *Fo-MYC2*) at 500 µg ml^−1^ to L1 larvae. (**c**) Effect of two MYC orthologs on expressions of EpOME synthetic (=*Fo-CYP24*) and degrading (=*Fo-sEH2*) genes. Each dsRNA was fed to L1 larvae at 500 µg ml^−1^. After 12 h, the L1 larvae were infected with TSWV. The expression levels of *Fo-CYP24* and *Fo-sEH2* were assessed at 24 h after the viral infection, at which the viral titres were assessed. (**d**) Upregulation of an E3 ubiquitin-protein ligase, UBR7, by EpOME via two MYC orthologues. L1 larvae were fed with TSWV or 12,13-EpOME at 0.1 µg ml^−1^. ‘CON’ represents naïve larvae. For RNAi treatments of *Fo-CYP24* and *Fo-sEH2* expressions, L1 larvae were fed with individual gene-specific dsRNAs (dsCYP24 to *Fo-CYP24* and dssEH2 to *Fo-sEH2*) at 500 µg ml^−1^. The expression levels of *UBR7* were assessed at 24 h after the emergence of adult thrips that had been treated during their L1 larval stage by TSWV or EpOME. (**e**) A schematic diagram of control of EpOME level by NSs via two MYC orthologs to suppress antiviral response in *F. occidentalis*.

To further investigate the influence of TSWV on the MYC/MAX expression, RNAi treatments targeting individual TSWV genes (*N*, *NSm*, *NSs* and *RdRP*) were applied to TSWV-infected thrips (Fig. 5a). Four different RNAi treatments significantly suppressed *Fo-MAX* expression, with the highest suppression observed in dsRNA^NSm^ and dsRNA^NSs^ treatments. Interestingly, *Fo-MYC* expression was significantly downregulated only by dsRNA^NSm^ and dsRNA^NSs^ treatments. To assess the functional role of these transcription factors in EpOME regulation, RNAi treatments specific to *Fo-MYC* (dsMYC) and *Fo-MAX* (dsMAX) were applied. Both treatments suppressed gene expression by more than 50% for at least 48 h post-treatment (Fig. 5b). In TSWV-infected larvae, the individual RNAi treatments against either *Fo-MYC* or *Fo-MAX* prevented the upregulation of the EpOME synthase gene (*Fo-CYP24*) and the downregulation of the degradation gene (*Fo-sEH2*), compared to untreated controls (Fig. 5c). Moreover, TSWV titres were also significantly reduced in larvae treated with either dsMYC or dsMAX, confirming the involvement of these transcription factors in supporting viral replication.

UBR7, an E3 ubiquitin-protein ligase, has been shown to play a key role in facilitating TSWV multiplication in *F. occidentalis* [44]. As expected, TSWV infection significantly induced *UBR7* expression in adult thrips. Interestingly, EpOME injection alone also induced *UBR7* expression in *F. occidentalis* (Fig. 5d). Conversely, RNAi-mediated silencing of *CYP24* reduced *UBR7* expression under TSWV infection, while silencing *sEH2* enhanced its expression. This suggests that elevated EpOME levels during TSWV infection contribute to UBR7 upregulation and viral replication (inset diagram in Fig. 3a). Furthermore, silencing *Fo-MYC* or *Fo-MAX* significantly reduced *UBR7* expression in TSWV-infected adults. Collectively, our findings provide a working model in which TSWV manipulates host immunity via its NSs protein (Fig. 5e). NSs is proposed to activate the MYC/MAX complex, which could in turn modulate expression of the EpOME biosynthetic gene *Fo-CYP24* and the degradation enzyme *Fo-sEH2*, resulting in elevated EpOME levels. EpOMEs, potentially through an unknown receptor, inhibit secondary messenger levels such as cAMP and calcium signalling, leading to suppression of antiviral response. Simultaneously, EpOMEs enhance *UBR7* expression, further suppressing antiviral defenses and facilitating viral replication and immune evasion during TSWV infection.

### Polymorphic loci in the NSs sequence between RB and WT TSWV strains against hot pepper

**Fig. 6.**
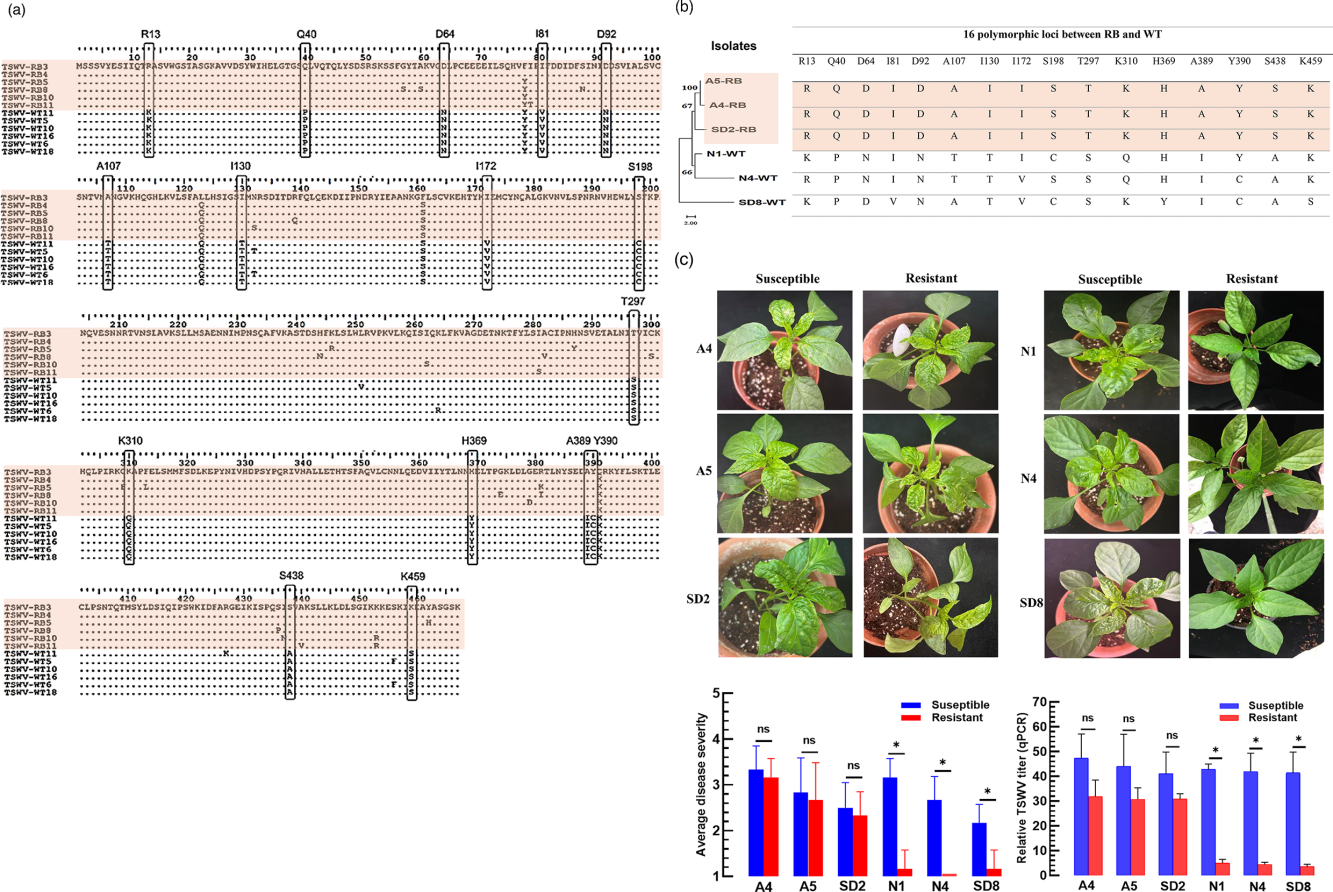
Polymorphic loci in the *NSs* gene characteristic of RB strains of TSWV against hot peppers. (**a**) Multiple alignment of 12 *NSs* amino acid sequences from 6 RB and 6 WT strains using mega 11 software. Dichotomous amino acid residues are shown in the boxes. Their GenBank accession numbers are shown in Table S3. (**b**) A phylogenetic analysis of six TSWV isolates (Table S4) in NSs amino acid sequences and their 16 dichotomous loci. (**c**) Disease symptoms of the six TSWV isolates against resistant (*C. annuum* cv. Color-Jjang) and susceptible (*C. annuum* cv. Chungyang) hot pepper cultivars. Fourteen days post-inoculation, host symptoms were evaluated by disease severity and assessed in relative TSWV titres. An asterisk above standard error bars indicates a significant difference between susceptible and resistant varieties at type I error=0.05 (LSD test). ‘NS’ represents no significant difference.

To identify polymorphic loci in the amino acid residues between RB and WT TSWV strains, six isolates from each group were compared based on their predicted sequences (Fig. 6a). Sixteen polymorphic loci clearly distinguished RB strains from WT strains. NSs amino acid sequences of six TSWV isolates identified in this study from symptomatic hot peppers were compared (Fig. 6b). In a phylogenetic analysis, the three RB strains were distinctly clustered apart from WT strains. Furthermore, the 3 RB strains shared all 16 RB-specific polymorphic loci, while some WT strains occasionally exhibited RB-specific residues at eight of these loci.

To confirm RB and WT strains in host plants, the isolates were mechanically inoculated into both TSWV-resistant and susceptible hot pepper cultivars (Fig. 6c). All six strains induced characteristic disease symptoms in the susceptible cultivar. However, only RB strains showed disease symptoms in resistant hot peppers. Comparison of disease severity and relative viral titres among the six isolates in susceptible and resistant hot peppers revealed significantly lower disease severity and viral accumulation for WT strains in resistant hot peppers.

### Differential control of EpOME biosynthesis of *F. occidentalis* by RB and WT TSWV strains

**Fig. 7.**
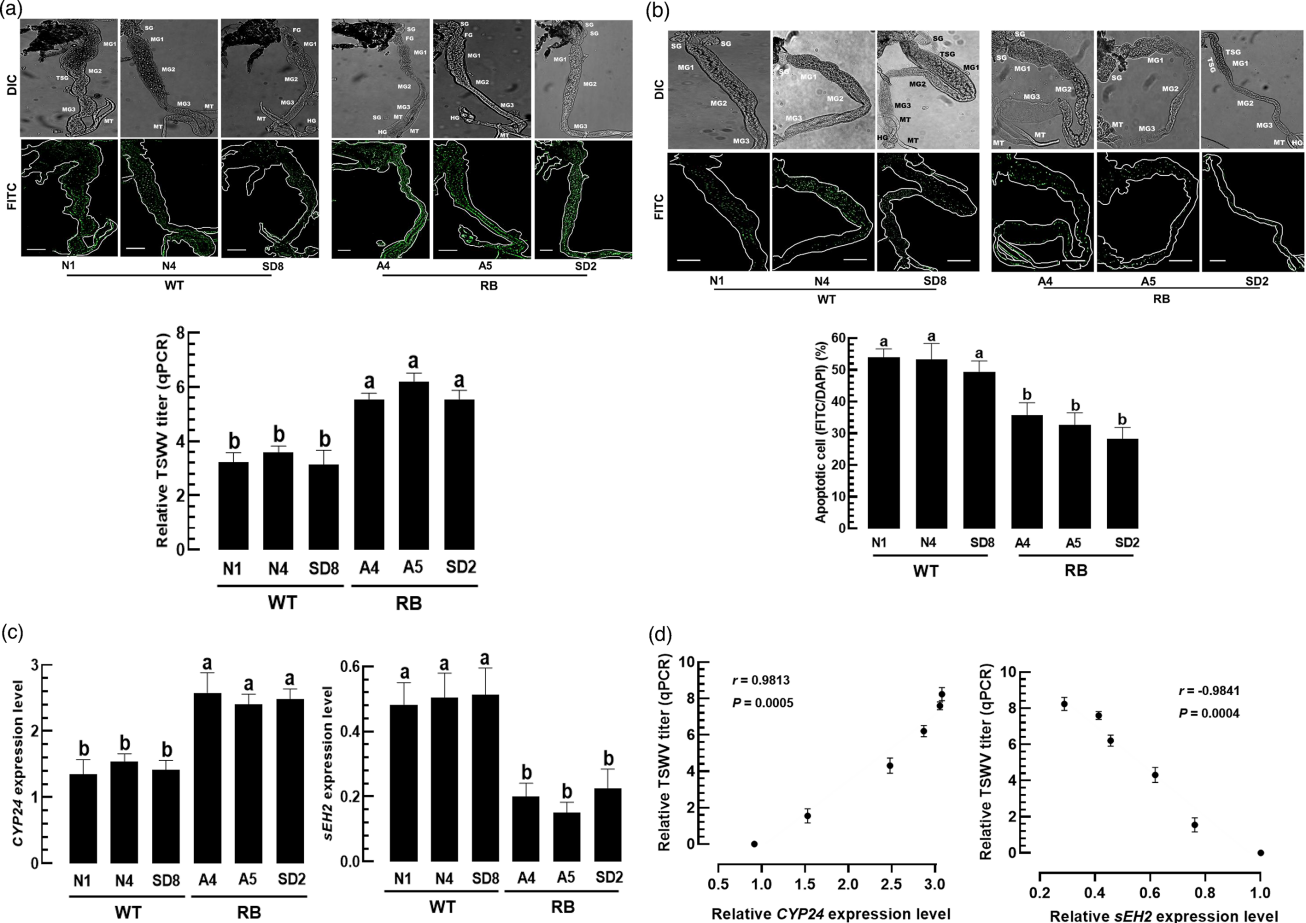
Differential control of TSWV titres in *F. occidentalis* larvae infected with RB and WT TSWV strains: SD2, A4 and A5 strains for RB and SD8, N1 and N4 strains for WT. These six different TSWV strains were fed to L1 larvae. After 24 h, TSWV, apoptosis and gene expression were analysed. (**a**) Variation in TSWV titre in the thrips. The viral particles in the gut epithelium were visualized by fluorescence in situ hybridization assay. The tissues and virus were visualized using differential interference contrast (DIC) and fluorescein isocyanate (FITC), respectively, under a fluorescent microscope (DM2500, Leica, Wetzlar, Germany). Scale bars represent 0.1 mm. Relative TSWV litres were assessed by real-time qPCR. Each treatment was replicated three times. (**b**) Variation in apoptosis in the gut epithelium of the thrips. Apoptosis was performed by TUNEL assay with an anti-mouse IgG specific to BrdU. FITC-positive cells were counted among randomly selected 100 cells stained by DAPI. (**c**) Variation of different TSWV strains in modulating expressions of EpOME synthetic (=*Fo-CYP24*) and degrading (=*Fo-sEH2*) genes. Different letters above standard deviation bars indicate significant differences among means at type I error=0.05 (LSD test). (**d**) Regression pattern between TSWV titre and the expression level of *CYP24* and *sEH2*.

To assess the influence of NSs sequence variation between RB and WT strains on TSWV multiplication in *F. occidentalis*, feeding inoculation was performed. TSWV viral particles were clearly abundant in the midgut of virus-infected thrips larvae (Fig. 7a). Interestingly, thrips fed with RB strains harboured more viral particles than those fed with WT strains. Using real-time qPCR, the viral titres were quantitatively assessed and showed that the RB strains showed significantly more viral titres than the WT strains in the thrips gut.

To explain the observed differences in viral titres, apoptosis in infected thrips was analysed using the TUNEL assay (Fig. 7b). All TSWV-infected larvae exhibited a positive signal in the TUNEL assay, indicating apoptosis in the infection foci. Compared to RB strains, the WT TSWV isolates induced significantly higher levels of apoptosis signals.

Given that EpOME suppresses antiviral responses, expression levels of EpOME biosynthetic and degradative genes in *F. occidentalis* were analysed after infection with RB and WT strains (Fig. 7c). RB strains significantly upregulated *CYP24* expression compared to WT strains. In contrast, WT strains more strongly induced the expression levels of *sEH2* compared to RB strains. These data revealed a strong positive correlation between TSWV titre and the *CYP24* expression level and a strong negative correlation between TSWV titre and *sEH2* expression (Fig. 7d).

### Differential control of antiviral responses of *F. occidentalis* by RB and WT TSWV strains

**Fig. 8.**
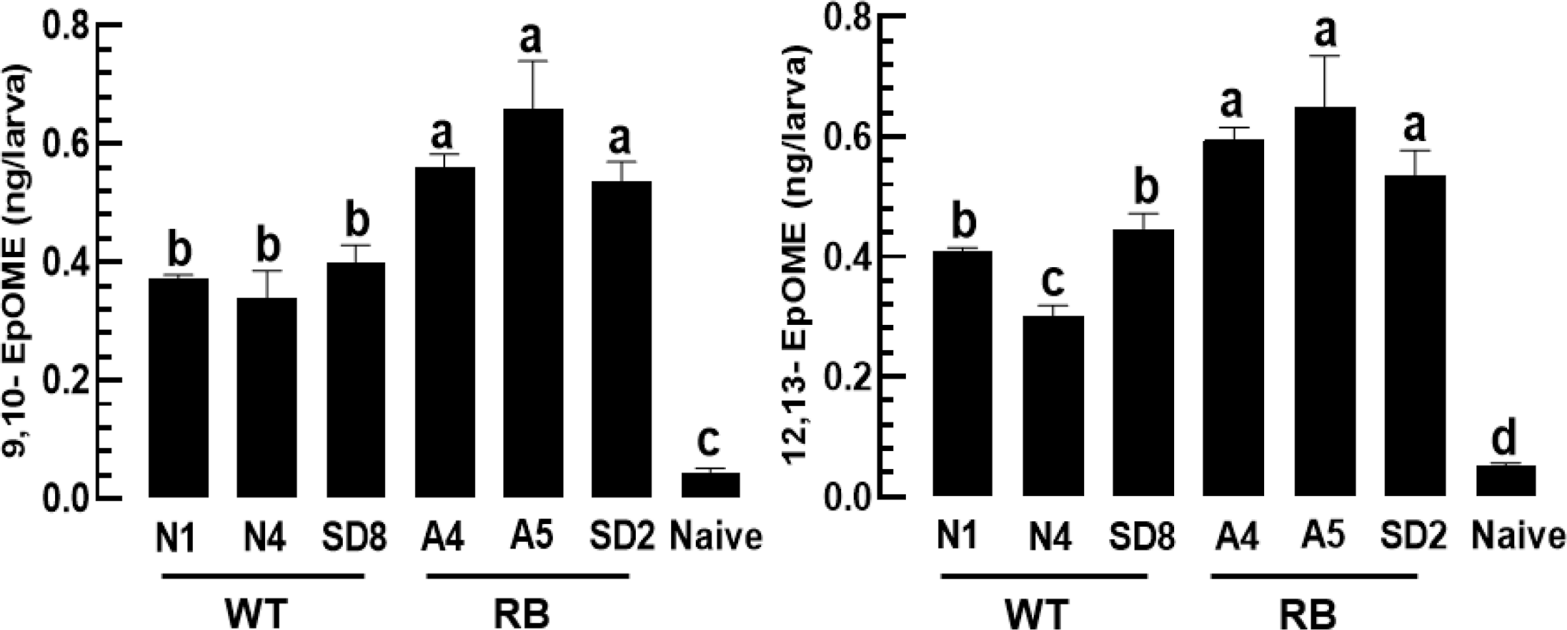
Differential control of EpOME titres in *F. occidentalis* larvae infected with RB and WT TSWV strains: SD2, A4 and A5 strains for RB and SD8, N1 and N4 strains for WT. These six different TSWV strains were fed to L1 larvae. After 24 h, EpOME levels were analysed using LC-MS/MS. See the raw data in Fig. S1. Quantification of two EpOMEs in the virus-infected larvae compared to naïve larvae. Each treatment was conducted in triplicate. Different letters above standard deviation bars indicate a significant difference among means at type I error=0.05 (LSD test).

**Fig. 9.**
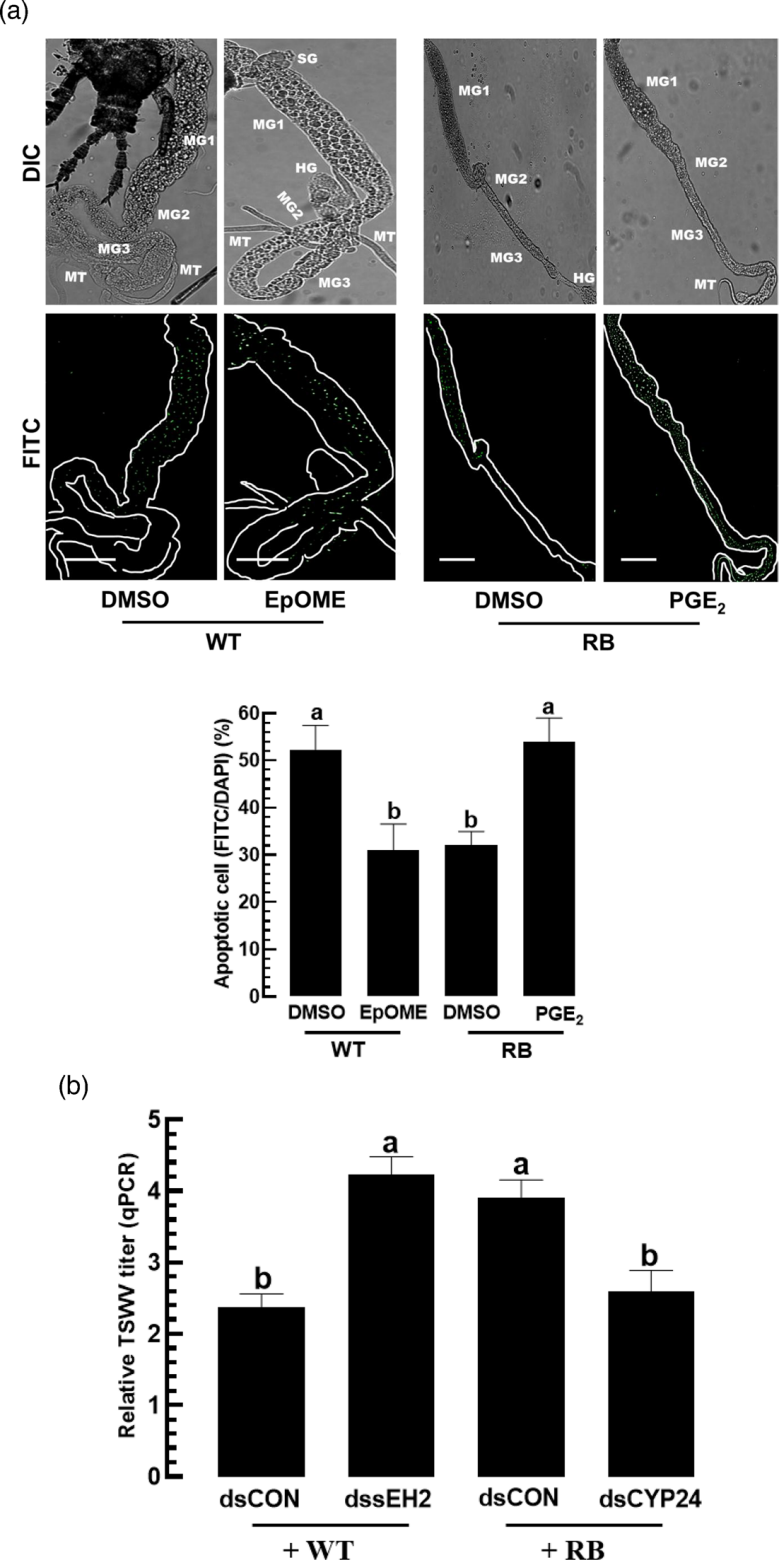
Interconversion between RB (A4) and WT (N1) TSWV strains in their viral titres by manipulating EpOME levels in *F. occidentalis*. (**a**) Influence of 12,13- EpOME (0.1 µg ml^−1^) or PGE_2_ (10^−7^ M) addition to RB or WT strain on their phenotypic conversion in an antiviral response measured by apoptosis in the gut epithelium of the thrips larvae using TUNEL assay with an anti-mouse IgG specific to BrdU under a fluorescent microscope (DM2500, Leica, Wetzlar, Germany). The whole tissues and the apoptotic cells were observed by differential interference contrast (DIC) and fluorescein isocyanate (FITC) modes, respectively. FITC-positive cells were counted among randomly selected 100 cells stained by DAPI. Scale bars represent 100 µm. (**b**) Influence of RNAi specific to EpOME synthetic (=*Fo-CYP24*) and degrading (=*Fo-sEH2*) genes on the phenotypic conversion of RB and WT strains in their viral titres in *F. occidentalis*. For RNAi treatments of *Fo-CYP24* and *Fo-sEH2* expressions, L1 larvae were fed with individual gene-specific dsRNAs (dsCYP24 to *Fo-CYP24* and dssEH2 to *Fo-sEH2*) at 500 µg ml^−1^. Different letters above standard error bars indicate significant differences among means at type I error=0.05 (LSD test).

To support the differential control of EpOME biosynthesis by RB and WT strains, EpOME levels were measured in thrips infected with either strain type using LC-MS/MS (Figs 8, S1). As expected, thrips infected with RB strains had significantly higher levels of 9,10-EpOME and 12,13-EpOME than the thrips infected with the WT strain. These findings suggest that manipulation of the EpOME level in thrips could alter the RB or WT status of the virus. To test this hypothesis, EpOME was added during WT strain infection (Fig. 9a), which significantly suppressed apoptosis. In contrast, PGE_2_ addition to the RB strain infection significantly increased the antiviral response. Furthermore, EpOME biosynthesis was manipulated by RNAi targeting *sEH2* or *CYP24* (Fig. 9b). RNAi specific to *sEH2* expression increased viral titres in WT-infected thrips, whereas RNAi treatment of *CYP24* expression decreased viral titres in RB-infected thrips.

### Differential control of insect immune responses by RB and WT TSWV strains

**Fig. 10.**
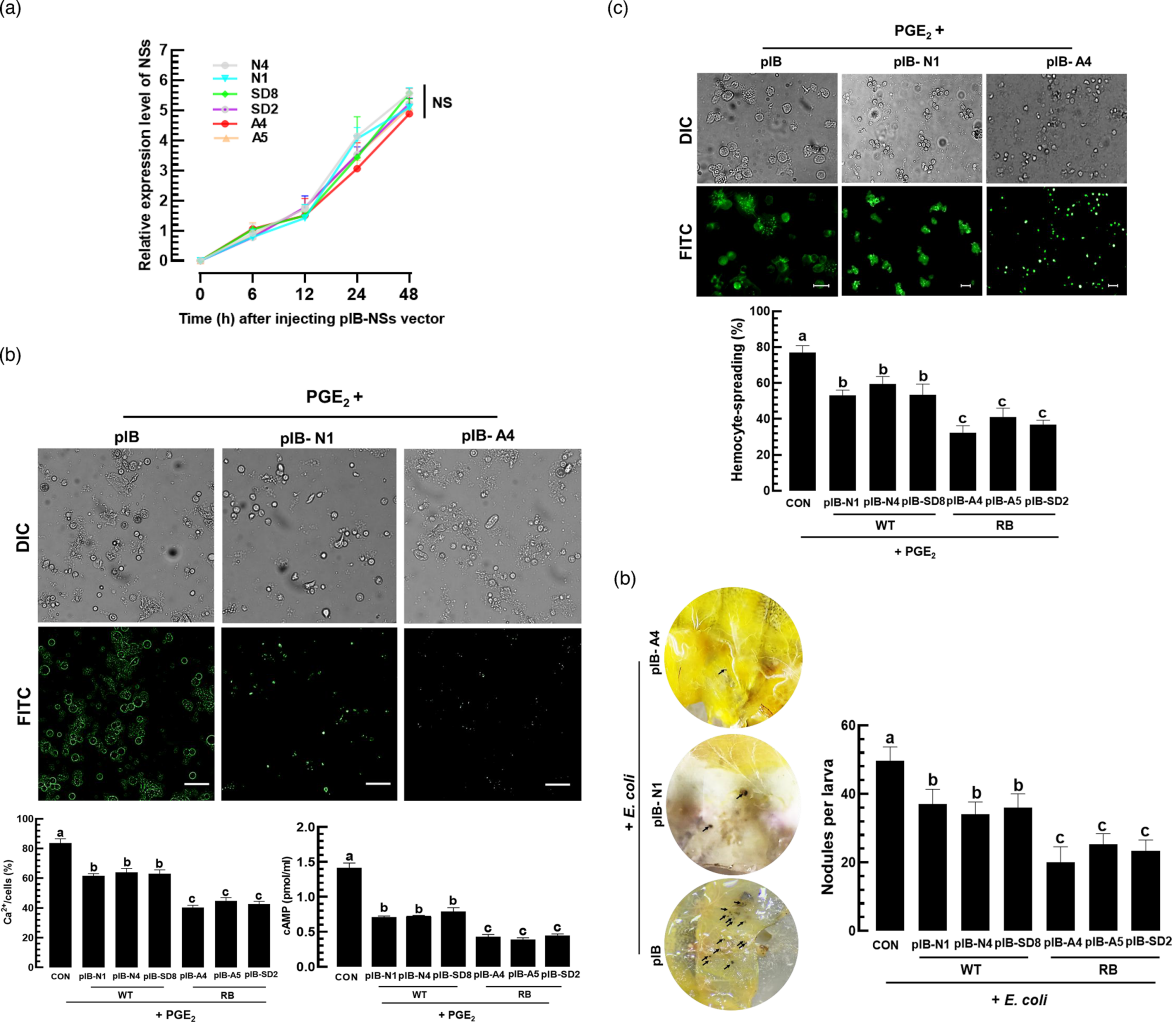
Differential control of insect immune responses by different *NSs* genes derived from RB and WT TSWV strains in a lepidopteran insect, *S. exigua*: SD2, A4 and A5 strains for RB and SD8, N1 and N4 strains for WT. Different NSs genes were cloned into an expression vector, pIB, under a baculoviral immediately early (IE) promoter and used for IVTE in *S. exigua* larvae. (**a**) IVTE of six NSs genes in *S. exigua* by injecting the recombinant pIB vector (200 ng/larva) into L5 larvae. Controls were injected with empty pIB (200 ng). (**b**) Differential control of secondary messenger (cAMP and Ca^2+^) levels in the haemocytes by the NSs-IVTE. Cells were incubated with 1 µL of PGE_2_ (10^−7^ M) for 40 min. FITC-positive cells were counted among randomly selected 100 cells stained by DAPI. Quantification of cAMP used a cyclic AMP ELISA kit (Cayman Chemical, Ann Arbor, MI, USA). Scale bars indicate 10 µm. (**c**) Effects of NSs-IVTE on haemocyte-spreading behaviour. At 24 h post-IVTE injection, haemocytes were collected and incubated with 1 µL of PGE_2_ (10^−7^ M) for 40 min, and spreading behaviour was quantified by counting the cells showing F-actin growth (stained by FITC) beyond cell boundaries among 100 randomly chosen cells. (**d**) Effects of NSs-IVTE on nodule formation. At 24 h post-IVTE injection, the larvae were injected with *E. coli* (2×10^4^ cells per larva). After 8 h, the nodules were counted in the haemocoel of the larvae. Each experiment was replicated three times. Each treatment was replicated three times. Different letters above standard error bars indicate significant differences among means at type I error=0.05 (LSD test).

To evaluate how *NSs* expression influences insect immune responses, cellular immune responses of *S. exigua* were assessed by IVTE. Haemocoelic injection of expression vectors carrying different *NSs* sequences confirmed uniform expression levels (Fig. 10a). Under this transient expression of *NSs*, secondary messenger levels in haemocytes were compared (Fig. 10b). PGE_2_ injection upregulated cAMP and Ca^2+^ levels. However, the *NSs* expression suppressed the secondary messenger levels, in which RB-derived *NSs* were more potent than WT-derived *NSs*. Haemocyte-spreading behaviour of *S. exigua* was stimulated by PGE_2_ treatment (Fig. 10c), but suppressed by *NSs* expression, again with RB-derived *NSs* having a stronger effect. Haemocyte nodule formation was also suppressed by the *NSs* expressions (Fig. 10d), with *NSs* from RB strains being more effective than those from WT strains.

## Discussion

TSWV causes an uncontrolled disease in hot peppers and other economically important crops. Although *Tsw* gene-based resistant cultivars have been widely cultivated, the emergence of resistance-breaking TSWV strains, primarily due to point mutations in the *NSs* gene, has reduced their effectiveness [9]. In addition to their virulence in plants, our study demonstrates the outstanding replication of the RB strains in the vector insect, *F. occidentalis*. Building on our previous findings [32] that NSs can induce the production of EpOME in *F. occidentalis*, as it mediates antiviral response, this study showed that the RB-type *NSs* induce more EpOME than the WT strains.

Two oxylipins antagonistically regulate TSWV titres in *F. occidentalis* by modulating antiviral response measured through apoptosis in the gut. While PGE_2_ stimulated apoptosis in the gut and reduced viral titres, EpOME suppressed apoptosis and enhanced viral replication. Antiviral responses mediated by PGE_2_ are well established in metazoans. For example, the infectious bronchitis virus (IBV) in chicks is an enveloped RNA virus that causes deformities in eggshells. An attenuated IBV infection induces a series of innate immune responses, including the expression of viral RNA recognition receptors and innate antiviral factors, along with an increase in PGE_2_ [45]. The study also demonstrated the direct role of PGE_2_ in mediating the expression of RNA virus pattern recognition receptors (TLR3, TLR7 and MDA5), antimicrobial peptides and interferons. TSWV infection in *F. occidentalis* induces the genes associated with eicosanoid biosynthesis, which leads to PGE_2_ production [24]. Kim *et al*. [17] explained the upregulation of eicosanoid production in *F. occidentalis* upon TSWV infection by stimulating the release of damage-associated molecules from the infection foci of the larval gut, which in turn stimulated phospholipase A_2_ for eicosanoid biosynthesis. In contrast, EpOME acts as a negative regulator of the antiviral immunity in insects [29]. Shahmohammadi *et al*. [31] demonstrated the role of EpOME by elevating the virulence of an insect pathogenic baculovirus against *S. exigua* and other lepidopteran insects by suppressing the expression of caspase-dependent apoptosis. Thus, the antagonistic actions of these two oxylipins can be understood in the regulation of TSWV viral titres in *F. occidentalis*.

The antagonistic actions of the two oxylipins were also demonstrated in the secondary messenger levels in the gut epithelium of *F. occidentalis*. Upon TSWV infection, cAMP and Ca^2+^ levels were elevated in the gut epithelium, in which PGE_2_ addition further elevated these levels, while EpOME addition suppressed them. The modulation of the secondary messenger levels can be explained by the intracellular signalling of PGE_2_ via its specific receptor, PGE_2_ receptor (PGE_2_R). Insect PGE_2_R was first identified in two lepidopteran insects, *S. exigua* and *Manduca sexta* [7,46], and functionally associated with two different types of trimeric Gα protein: Gαs and Gα(12) elevating secondary messenger levels of cAMP and Ca^2+^ [47]. The upregulation of these two secondary messenger levels in response to PGE_2_ in *F. occidentalis* indicates that the thrips may possess a similar type of PGE_2_R and its trimeric G proteins. These findings suggest that EpOME suppressed the secondary messenger levels and reduced the apoptosis in the thrips gut to protect TSWV from the antiviral response.

TSWV upregulated EpOME levels in *F. occidentalis* to suppress the antiviral response. The viral infection induced the expression of *CYP24* for EpOME biosynthesis while it suppressed *sEH2* expression to prevent degradation of the endogenous EpOMEs. The addition of AUDA (a specific inhibitor of sEH enzyme activity) significantly increased the viral load in the infected thrips. Among five different genes encoded in the TSWV genome, *NSs* is responsible for the elevation of EpOME in *F. occidentalis* [32]. This explains the suppression of TSWV load after the RNAi specific to *NSs* expression in the virus-infected thrips. Alternatively, a transient expression of the viral *NSs* gene in *S. exigua* modulated the genes associated with EpOME synthesis and degradation. This transient expression was demonstrated by *EGFP* expression, supporting the IVTE technique [39].

NSs upregulated EpOME level in *F. occidentalis*, potentially through interaction with a transcription factor, *Fo-MYC2*, which modulates the genes associated with EpOME synthesis and degradation. *Drosophila* MYC (dMYC) is a transcription factor that mediates diverse cellular processes, such as cell cycle progression, cell growth, proliferation and apoptosis. In addition, it acts as a negative regulator of innate immunity by inhibiting IMD immune signalling via directly activating *miR-277* transcription [48]. This is vital to restore immune homeostasis after a burst of immune responses upon pathogen infection.

Different TSWV isolates with varying *NSs* sequences were categorized as RB or WT strains. Polymorphisms in the NSs protein have been widely recognized as key determinants of the RB phenotype. Margaria *et al*. [13] provided compelling evidence that mutations in the NSs protein enable TSWV to overcome the *Tsw*-mediated resistance in pepper. Similarly, Almási *et al*. [49] reported increasing diversity among RB strains, supporting the notion that multiple loci in the *NSs* gene contribute to the ability of the virus to break host resistance. Moreover, Kwon *et al*. [50] demonstrated that reassortment events involving the S RNA segment – which encodes NSs – play a significant role in the emergence of RB variants, as segment exchanges can introduce novel polymorphisms. The variability in the reported polymorphic loci across different studies, including those by Margaria *et al*. [13], Almási *et al*. [49] and Debreczeni *et al*. [51], is likely due to geographical differences, host-specific adaptations and the evolutionary pressure exerted by the widespread cultivation of *Tsw*-resistant pepper cultivars. We identified 16 polymorphic loci distinguishing RB isolates from WT strains of TSWV, with particular emphasis on the D92 locus, which was consistently reported by de Ronde *et al*. [52]. This classification was further supported by symptomatic analysis using TSWV-susceptible and resistant hot peppers. However, a previously reported single point mutation associated with resistance breaking – T104A in the NSs protein [5] – was absent in all our isolates, consistent with findings by de Ronde *et al*. [52] and Almási *et al*. [49]. These observations reinforce the hypothesis that resistance-breaking in TSWV is a multifactorial trait involving diverse mutations rather than a universal mechanism. Altogether, our findings highlight the evolutionary plasticity of the *NSs* gene and underscore the importance of ongoing surveillance and molecular characterization of TSWV populations to effectively manage emerging RB strains in pepper cultivation systems. A limitation of this study is that S-segment reassortment was not specifically evaluated; however, we minimized potential misclassification by selecting well-characterized isolates and verifying their sequences prior to analyses.

Interestingly, RB strains upregulated EpOME biosynthesis more than WT strains. The high induction of EpOME biosynthesis of RB strains led to stronger suppression of the antiviral response compared to WT strains. Our results are consistent with reports that RB strains often spread more effectively than WT isolates under resistance pressure. RB isolates are transmitted by *F. occidentalis* with higher efficiency and may enhance vector performance such as greater fecundity and adult longevity, promoting their field dominance [53,54]. This difference in the immunosuppressive activities between RB and WT strains was further supported in the non-vector insect *S. exigua*, which exhibited different cellular immune responses depending on the NSs source. These results clearly indicate that NSs induce EpOME biosynthesis to suppress antiviral response, facilitating TSWV transmission by the insect vector. Worldwide genomic analysis of TSWV indicates that most (83%) isolates are geographically differentiated, but others are likely to have diverged following the initial introduction of *F. occidentalis*, particularly due to genetic variation in *NSs* associated with resistance breaking [55]. The introgression of *Tsw* resistance gene into the pepper cultivars was initially effective for TSWV control, but the widespread use of resistant cultivars evoked the rapid emergence of RB TSWV strains presumably with NSs acting as an avirulent factor against *Tsw*-mediated resistance [52]. The first RB strains were detected in hot pepper crops in Korea [56]. Our current study identified 16 polymorphic loci specific to RB strains compared to WT hot peppers. Some controversy may exist in these RB-specific loci, as RB isolates could evolve differentially depending on geographical variation and differential selection pressure [5]. From an evolutionary perspective, two non-exclusive pressures may contribute to the emergence of these polymorphisms. On the plant side, widespread deployment of *Sw-5* and *Tsw* resistance genes likely exerts strong selection favouring resistance-breaking variants. On the vector side, RB strains may be favoured if polymorphisms enhance transmission efficiency or vector fitness through immunomodulation. Finally, these findings suggest potential applied avenues, such as targeting EpOME or PGE₂ signalling pathways to disrupt virus-induced immune suppression in thrips. These implications remain speculative but highlight the importance of linking NSs molecular variation to epidemiological outcomes.

The differential modulation of EpOME production by RB and WT NSs provides insight into the variation in functional interaction between *NSs* and *Fo-MYC2*. A stronger induction of *Fo-MYC* expression by RB-derived *NSs* compared to WT-derived *NSs* leads to elevated EpOME production, which in turn strongly suppresses the antiviral response in thrips. This results in higher viral load in the insect vector, increasing the likelihood of successful transmission and disease establishment in hot pepper plants.

## Supplementary material

10.1099/jgv.0.002175 Uncited Supplementary Material 1.

## Abbreviations

AUDA: 12-(3-adamantan-1-ylureido) dodecanoic acid
BrdU: 5-bromo-2′-deoxyuridine
EpOMEs: epoxyoctadecamonoenoic acids
FISH: fluorescence *in situ* hybridization
IBV: infectious bronchitis virus
IVTE: *in vivo* transient expression
LSD: least significant difference
NSs: nonstructural protein S
PGE_2_R: PGE_2_ receptor
pi: post-injection
PUFAs: polyunsaturated fatty acids
qPCR: qualitative PCR
RB: resistance-breaking
RNAi: RNA interference
RT: room temperature
sEH: soluble epoxide hydrolase
SSC: saline sodium citrate
TSWV: tomato spotted wilt virus
