# Supplementary material for: Wild-type and resistance-breaking strains of tomato spotted wilt virus differentially upregulate the immunosuppressive epoxyoctadecamonoenoic acid biosynthesis of its insect vector, Frankliniella occidentalis
Source: J Gen Virol. 2025 Nov 7;106(11):002175. doi: 10.1099/jgv.0.002175 (PMC12594347; doi:10.1099/jgv.0.002175)
Supplement: Uncited Supplementary Material 1. [file jgv-106-02175-s001.pdf]

Supplementary Information

Table S1. Sequences of primers and probes used in this study and their PCR conditions

Table S2. Characteristics of tomato spotted wilt virus isolates identified in this study

Table S3. GenBank accession numbers of the tomato spotted wilt virus strains selected in this study for phylogenetic analyses

Table S4. GenBank accession numbers and abbreviations used for bioinformatic analysis of transcription factors in insects

Fig. S1. Raw data of LC-MS/MS measurements of 9,10-EpOME and 12,13-EpOME levels in *F. occidentalis* in response to different TSWV strains

Table S1. Sequences of primers and probes used in this study and their PCR conditions

Primer	Use	Orientation	Sequence (5'-3')	Annealing temperature (ºC)	Amplicon (bp)
EF1	qPCR	Forward	TCA AGG AAC TGC GTC GTG GAT	52	130
		Reverse	ACA GGG GTG TAG CCG TTA GAG		
Fo-UBR7	qPCR	Forward	GCAAGAGACCATATCCTGATCC	54	240
		Reverse	AGGGATCCTGATCCACTTCTAC		
Fo-CYP24	qPCR	Forward	CGCCGCACTCAAGAACT	54	304
		Reverse	CAAGAAGGGCAGGAAGTTGA		
Fo-sEH2	qPCR	Forward	TTCTTCCTGTTCCAAGTGCCTT	52	752
		Reverse	TTCTCATCCGGAGTGATTCCTTC		
Fo-MAX	qPCR	Forward	GATCTGTGATACGCACGTCTAAA	52	225
		Reverse	GGAGAGAAGCCTTGCTTGAATA		
Fo-MYC	qPCR	Forward	CGATCCAACTCAGCTGCTATAA	52	292
		Reverse	CGAAATTCCCAACGCTACATTC		
T7- Fo-MAX	RNAi	Forward	TAATACGACTCACTATAGGGAGAGA TCTGTGATACGCACGTCTAAA	52	275
		Reverse	TAATACGACTCACTATAGGGAGAGG AGAGAAGCCTTGCTTGAATA		
T7- Fo-MYC	RNAi	Forward	TAATACGACTCACTATAGGGAGACG ATCCAACTCAGCTGCTATAA	52	348
		Reverse	TAATACGACTCACTATAGGGAGACG AAATTCCCAACGCTACATTC		
TWSV-N	qPCR	Forward	ATGTCTAAGGTTAAGCTCACTAAGG AA	52	777
		Reverse	TTAAGCAAGTTCTGCAAGTATTGCC TG		
TWSV-NSs	qPCR	Forward	GCCTGCATTCCAAACCATAAC	54	439
		Reverse	CCCTGGCAAAGTCTATCTTCC		
Full ORF-NSs	PCR	Forward	TTTCGATCCTGAAGCATATGCTT	54	1400
		Reverse	TCATTTTGATCCTGAAGCATATG		
β-Actin	qPCR	Forward	CATCACCATCGGAAACGAAAGG	52	250
		Reverse	ATACTGTGTTGGCGTACAGGTC		

Table S2. Characteristics of tomato spotted wilt virus isolates identified in this study

Strain	Abbreviation	Origin	Accession number
TSWV-RB	TSWV-A5	Yunsang	PV472197
TSWV-RB	TSWV-A4	Yunsang	PV472198
TSWV-RB	TSWV-SD2	Sundong	PV472199
TSWV-WT	TSWV-N1	Songcheon	PV472200
TSWV-WT	TSWV-N4	Songcheon	PV472201
TSWV-WT	TSWV-SD8	Sundong	PV472202

Table S3. GenBank accession numbers of the tomato spotted wilt virus strains selected in this study for phylogenetic analyses

Strain	Abbreviation	Accession number
TSWV-RB	TSWV-RB3	UKD60018
TSWV-RB	TSWV-RB4	APG79475
TSWV-RB	TSWV-RB5	ABD38690
TSWV-RB	TSWV-RB8	QBB67470
TSWV-RB	TSWV-RB10	QGV12296
TSWV-RB	TSWV-RB11	QGV12294
TSWV-WT	TSWV-WT6	URC17583
TSWV-WT	TSWV-WT10	UKD60023
TSWV-WT	TSWV-WT11	QVW10150
TSWV-WT	TSWV-WT13	UYB94533
TSWV-WT	TSWV-WT15	AXA13023
TSWV-WT	TSWV-WT16	URC17577
TSWV-WT	TSWV-WT18	QPL18171

Table S4. GenBank accession numbers and abbreviations used for bioinformatic analysis of transcription factors in insects

**Protein**	**Accession number**	**Species**	**Abbreviation**
Se-Protein MAX	KAF9413100	*Spodoptera exigua*	Se-MAX1
Se-Protein MAX	CAH0690176	*S. exigua*	Se-MAX2
Se-Protein MAX	KAH9639207	*S. exigua*	Se-MAX3
protein MAX	XP-021202554	*Bombyx mori*	Bm-MAX
protein MAX	XP-623530	*Apis mellifera*	Am-MAX
Protein MAX	XP-026273402	*Frankliniella occidentalis*	Fo-MAX
Protein MAX	NP-001246833	*Drosophila melanogaster*	Dm-MAX
bHLH_transcription_factor_MYC	NP-001243937	*B. mori*	Bm-MYC
MYC_isoform_B	NP-001259204	*D. melanogaster*	Dm-MYC
transcriptional_regulator_MYC-B	XP-003250447	*A. mellifera*	Am-MYC
MYC protein	KAE8741034	*F. occidentalis*	Fo-MYC1
MYC protein	XP-052128859	*F. occidentalis*	Fo-MYC2
Basic helix-loop-helix (bHLH) DNA-binding family protein (MYC2)	NP-174541	*Arabidopsis thaliana*	At-MYC2

Analyte Name: 12,13-EPOME 1, 295.0 / 195.0

Internal Standard: N/A, N/A

**Data**		**Acquisition**	
**Data File**	2025-04-01 EET.wiff	**Method**	EET MRM5.dam
**Date and Time**	4/1/2025 10:01:45 PM	**Instrument**	QTRAP 4500 EB21491810
**Project**	Default	**Operator**	4500-PC\4500
		**Result Table**	2025-04-01 EET(PG)

Sample Name	Expected Concentration	Analyte Peak Area	IS Peak Area	Calculated Concentration	% Accuracy
10	10.0	2079	N/A	9.75	97.5
25	25.0	5758	N/A	25.04	100.2
50	50.0	11995	N/A	50.97	102.0
100	100.0	24174	N/A	101.61	101.6
200	200.0	47267	N/A	197.63	98.8

Regression Equation: y = 240.51662 x + -265.12482 (r = 0.99984, r^2 = 0.99967) (weighting: 1 / x), r^2=1.000

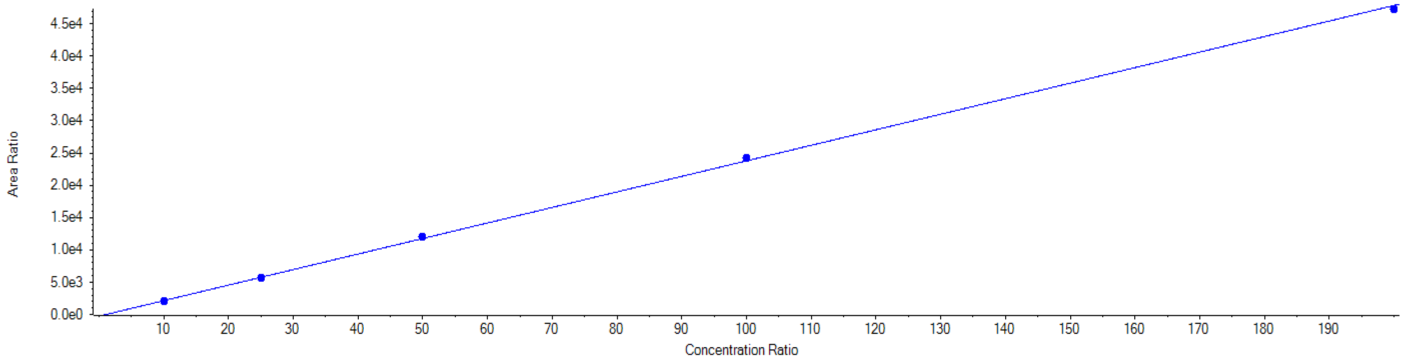

Sample Name	Analyte Peak Area	Analyte RT (min)	IS Peak Area	**Calculated Concentration (ng/mL)**
N1-1	145166	14.91	N/A	604.66
N1-2	146891	14.91	N/A	611.83
N1-3	149604	14.91	N/A	623.11

Sample Name	Analyte Peak Area	Analyte RT (min)	IS Peak Area	**Calculated Concentration (ng/mL)**
S2-1	205949	14.91	N/A	857.38
S2-2	195564	14.91	N/A	814.20
S2-3	174955	14.91	N/A	728.52
A4-1	221628	14.91	N/A	922.57
A4-2	213966	14.90	N/A	890.71
A4-3	205965	14.90	N/A	857.45
SD8-1	169652	14.91	N/A	706.47
SD8-2	149749	14.91	N/A	623.72
SD8-3	209383	14.91	N/A	671.66
A5-1	205162	14.91	N/A	854.11
A5-2	229792	14.91	N/A	956.51
A5-3	266495	14.92	N/A	1109.11
N4-1	181575	14.92	N/A	478.49
N4-2	102628	14.92	N/A	427.80
N4-3	161251	14.92	N/A	453.54

10

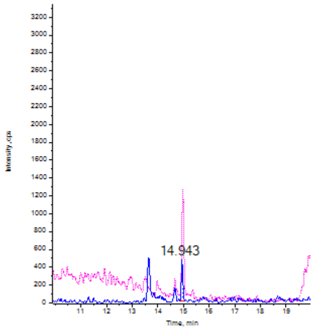

25

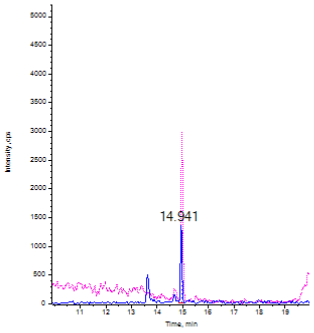

50

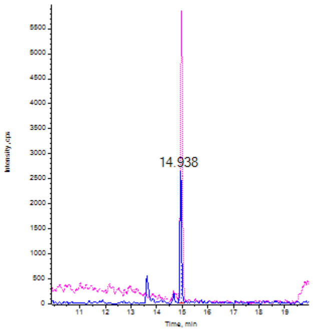

100

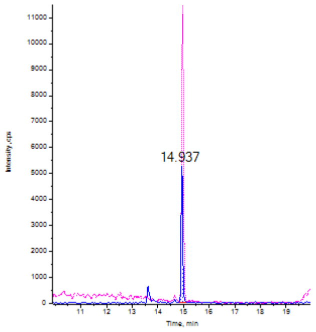

200

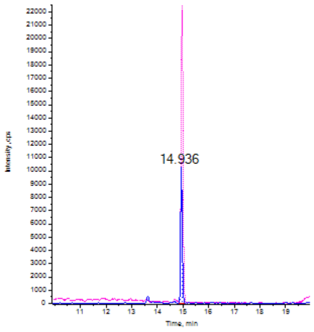

N1-1

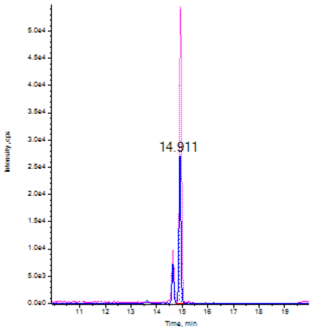

N1-2

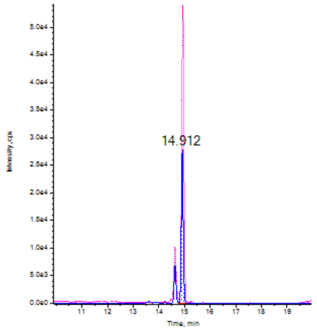

N1-3

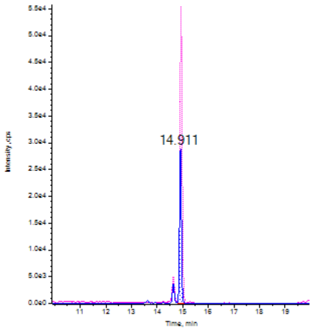

S2-1

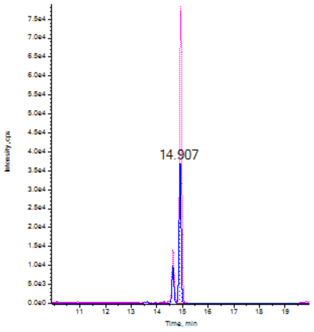

S2-2

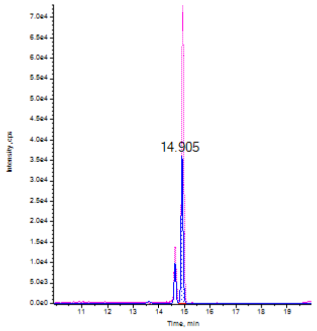

S2-3

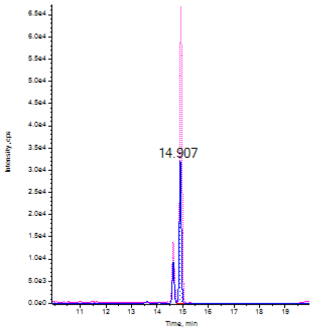

A4-1

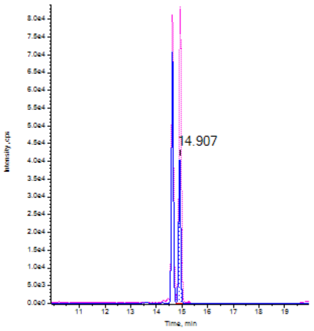

A4-2

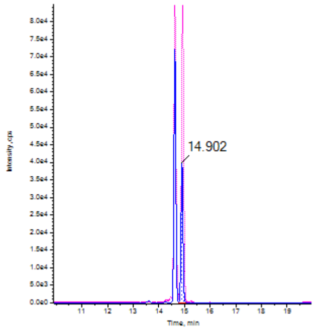

A4-3

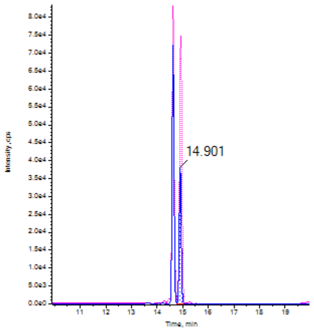

SD8-1

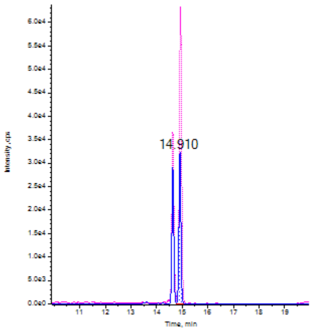

SD8-2

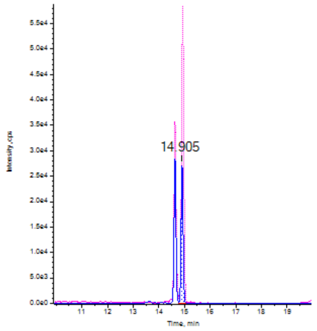

SD8-3

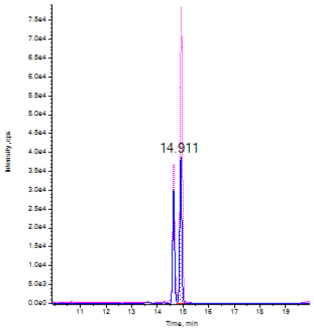

A5-1

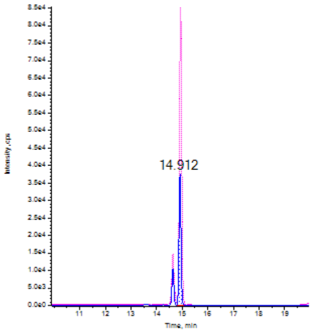

A5-2

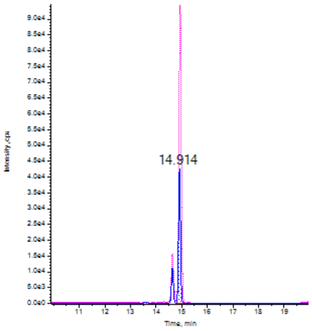

A5-3

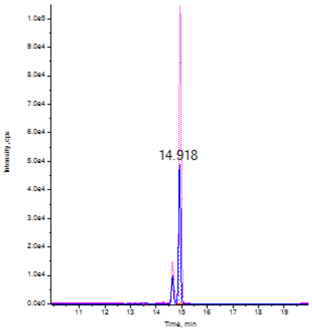

N4-1

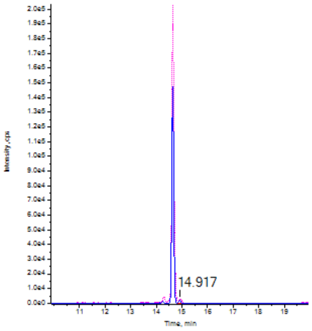

N4-2

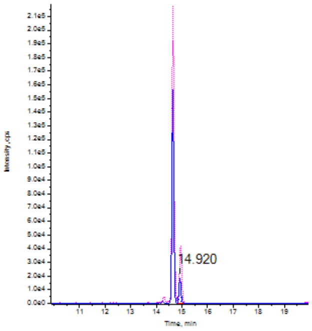

N4-3

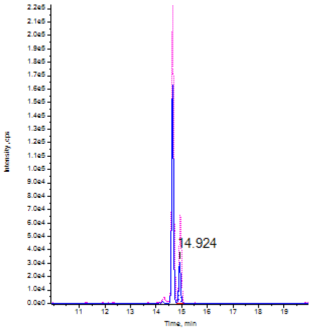

Analyte Name: 9,10-EPOME 1, 295.0 / 276.9

Internal Standard: N/A, N/A

**Data**		**Acquisition**	
**Data File**	2025-04-01 EET.wiff	**Method**	EET MRM5.dam
**Date and Time**	4/1/2025 10:01:45 PM	**Instrument**	QTRAP 4500 EB21491810
**Project**	Default	**Operator**	4500-PC\4500
		**Result Table**	2025-04-01 EET(PG)

Sample Name	Expected Concentration	Analyte Peak Area	IS Peak Area	Calculated Concentration	% Accuracy
10	10.0	8117	N/A	9.98	99.8
25	25.0	20660	N/A	25.40	101.6
50	50.0	40406	N/A	49.66	99.3
100	100.0	80254	N/A	98.63	98.6
200	200.0	163818	N/A	201.33	100.7

Regression Equation: $y = 813.69410\ x + -3.29839$ ($r = 0.99993$, $r^2 = 0.99985$) (weighting: 1 / x), r^2=1.000

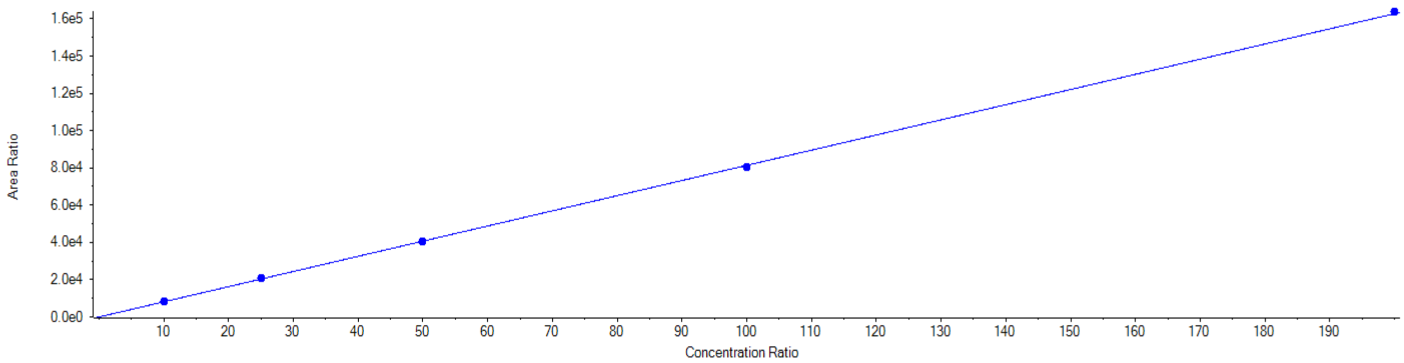

Sample Name	Analyte Peak Area	Analyte RT (min)	IS Peak Area	**Calculated Concentration (ng/mL)**
N1-1	461305	14.93	N/A	566.93
N1-2	456761	14.93	N/A	561.35
N1-3	444817	14.93	N/A	546.67

Sample Name	Analyte Peak Area	Analyte RT (min)	IS Peak Area	**Calculated Concentration (ng/mL)**
S2-1	656268	14.93	N/A	806.53
S2-2	611860	14.93	N/A	751.96
S2-3	572190	14.93	N/A	850.20
A4-1	712644	14.93	N/A	875.82
A4-2	682179	14.92	N/A	838.38
A4-3	660852	14.92	N/A	812.17
SD8-1	524386	14.93	N/A	644.46
SD8-2	486869	14.93	N/A	598.35
SD8-3	694755	14.93	N/A	853.83
A5-1	707180	14.93	N/A	869.10
A5-2	797053	14.94	N/A	979.55
A5-3	906331	14.94	N/A	1113.85
N4-1	426229	14.94	N/A	485.24
N4-2	352040	14.94	N/A	432.65
N4-3	559752	14.95	N/A	537.92

10

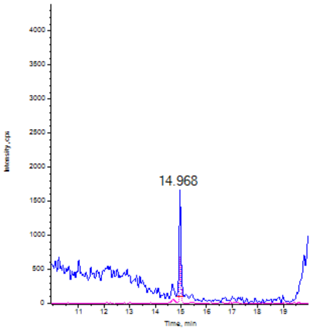

25

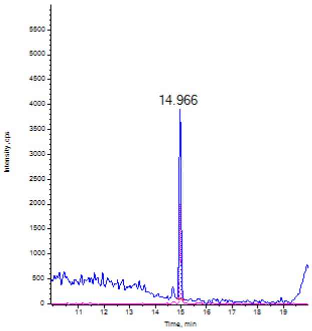

50

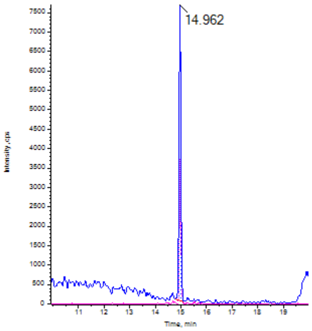

100

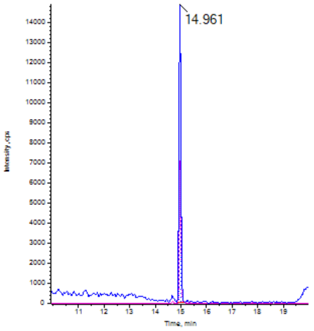

200

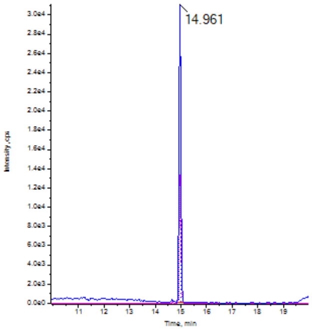

N1-1

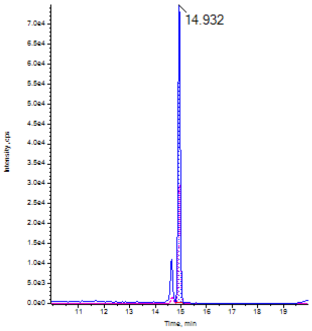

N1-2

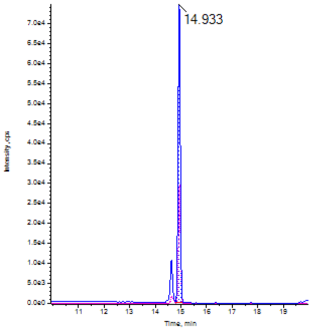

N1-3

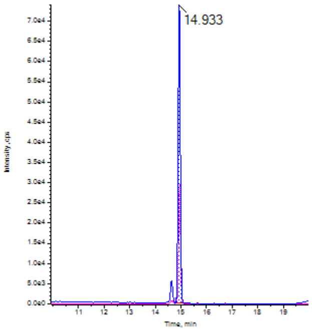

S2-1

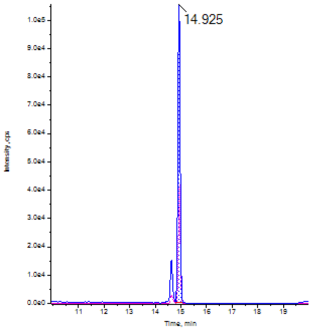

S2-2

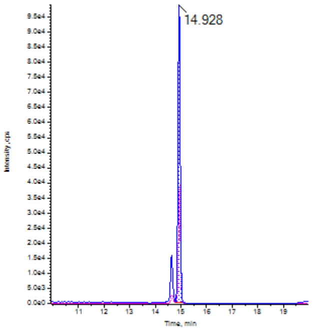

S2-3

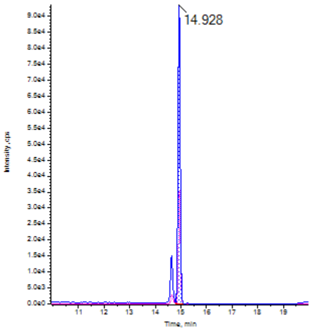

A4-1

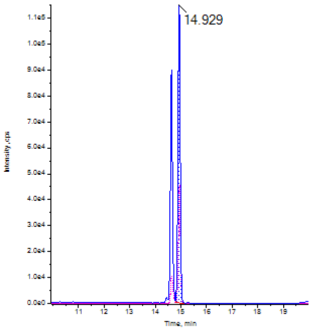

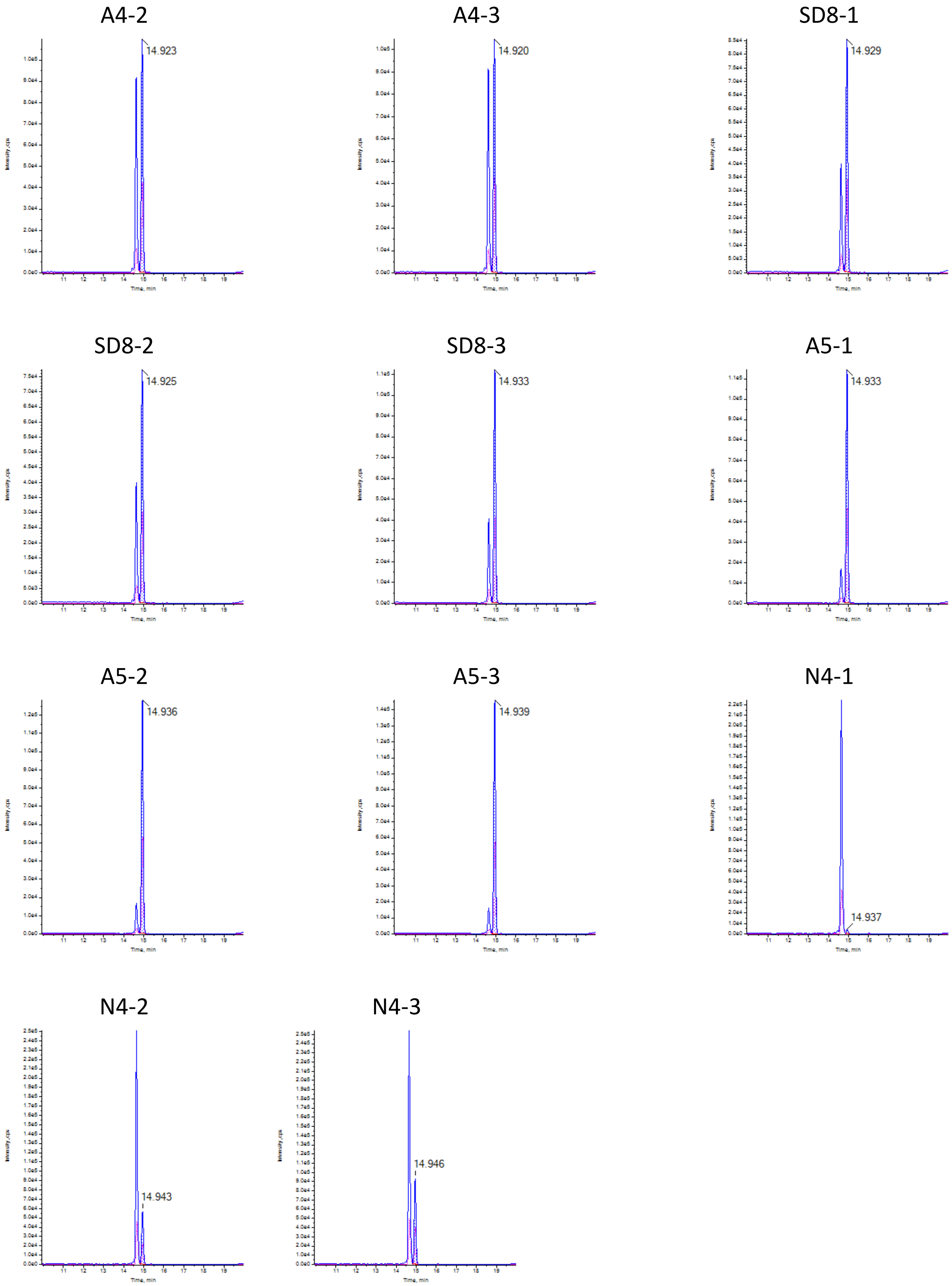
A4-2
14.923
A4-3
14.920
SD8-1
14.929
SD8-2
14.925
SD8-3
14.933
A5-1
14.933
A5-2
14.936
A5-3
14.939
N4-1
14.937
N4-2
14.943
N4-3
14.946

Fig. S1
